# Metabolic basis of neuronal vulnerability to ischemia; an *in vivo* untargeted metabolomics approach

**DOI:** 10.1038/s41598-020-63483-w

**Published:** 2020-04-16

**Authors:** Sherif Rashad, Daisuke Saigusa, Takahiro Yamazaki, Yotaro Matsumoto, Yoshihisa Tomioka, Ritsumi Saito, Akira Uruno, Kuniyasu Niizuma, Masayuki Yamamoto, Teiji Tominaga

**Affiliations:** 1 Department of Neurosurgical Engineering and Translational Neuroscience, Tohoku University Graduate School of Medicine, Sendai, Japan; 2 Department of Neurosurgery, Tohoku University Graduate School of Medicine, Sendai, Japan; 3 Department of Integrative Genomics, Tohoku Medical Megabank Organization, Sendai, Japan; 4 Medical Biochemistry, Tohoku University School of Medicine, Sendai, Japan; 5 Laboratory of Oncology, Pharmacy Practice and Sciences, Graduate School of Pharmaceutical Sciences, Tohoku University, Sendai, Japan; 6 Department of Neurosurgical Engineering and Translational Neuroscience, Graduate School of Biomedical Engineering, Tohoku University, Sendai, Japan

**Keywords:** Metabolomics, Cell death in the nervous system

## Abstract

Understanding the root causes of neuronal vulnerability to ischemia is paramount to the development of new therapies for stroke. Transient global cerebral ischemia (tGCI) leads to selective neuronal cell death in the CA1 sub-region of the hippocampus, while the neighboring CA3 sub-region is left largely intact. By studying factors pertaining to such selective vulnerability, we can develop therapies to enhance outcome after stroke. Using untargeted liquid chromatography-mass spectrometry, we analyzed temporal metabolomic changes in CA1 and CA3 hippocampal areas following tGCI in rats till the setting of neuronal apoptosis. 64 compounds in CA1 and 74 in CA3 were found to be enriched and statistically significant following tGCI. Pathway analysis showed that pyrimidine and purine metabolism pathways amongst several others to be enriched after tGCI in CA1 and CA3. Metabolomics analysis was able to capture very early changes following ischemia. We detected 6 metabolites to be upregulated and 6 to be downregulated 1 hour after tGCI in CA1 versus CA3. Several metabolites related to apoptosis and inflammation were differentially expressed in both regions after tGCI. We offer a new insight into the process of neuronal apoptosis, guided by metabolomic profiling that was not performed to such an extent previously.

## Introduction

Metabolomics is the study of metabolite composition of cells, tissues or biological fluids. Recent technological advances in this field has allowed the discovery of new biomarkers of diseases such as coronary artery disease, septic shock and brain tumors as well as the discovery of new drugs^1–5^. Metabolomic analysis has also deepened our understanding of several disease models, leading to the discovery of new pathways or targets for drug therapies that could later be applied to the treatment of various diseases^6–11^. Indeed, metabolomics are closer to the phenotype of a given disease compared with transcriptomic or proteomic analysis, both of which are prone to downstream modifications and changes in activities^1,12^.

Several techniques, tools and software platforms exist to study metabolomics, all of which complement each other^13^. The application of these tools has greatly enhanced our understanding of the pathophysiology of diseases in almost every field of research^14^. In the field of neuroscience, metabolomic analysis is used to study stroke^15^, brain tumors^16^, traumatic brain injury (TBI)^17,18^, neurodegenerative diseases^19^, hypoxic-ischemic encephalopathy^20^ and depression^21^ to name a few^22^. The application of metabolomic tools to study brain diseases have greatly enhanced our understanding of different pathologies, for example; we demonstrated the upregulation of the ceramide “Cer(d18:0/18:0)” and phosphocreatine following transient ischemia-reperfusion in mice using a mass spectrometric imaging approach^15^. Several studies have highlighted an array of small molecules, amino acids and lipids that were linked to stroke severity, progression or post-stroke cognitive deficits^23–25^. Moreover, linoleic acid metabolism, amino acid metabolism, galactose metabolism, and arachidonic acid metabolism were disturbed in the plasma of rats subjected to TBI^18^. Another study revealed a decrease of citrate and aconitate in TBI patients’ plasma amongst other disturbed metabolites^17^. Alzheimer’s disease (AD) and other neurodegenerative diseases are among the most studied by metabolomic approaches in the field of neuroscience^22^ and a magnitude of amino acids, energy metabolites, fatty acids, lipids and other metabolites were identified to be disturbed in AD^22,26–28^. The aforementioned examples clearly show the value of metabolomic analysis in enhancing our understanding of various diseases and how it enriched the field of neuroscience and led to new pathways and targeted therapies’ discovery.

Transient global cerebral ischemia (tGCI) model in rats is produced surgically by near total cessation of blood flow to the brain, inducing a state of global hypoxemia^6,29,30^, resulting in neuronal cell death in the CA1 sub-region of the hippocampus while other areas of the hippocampus are largely intact^6^. This contrast between the CA1 area and other areas of the hippocampus is particularly interesting when searching for pathways involved in neuronal vulnerability or resistance to ischemia, and can lead to the discovery of targets that can be used to modify the neuronal fate^6,31^. Moreover, the delayed nature of cell death after tGCI, contrary to focal ischemia models, allows the studying of pre-apoptosis events that cannot be otherwise studied^6^. Several mechanisms were discovered to be responsible for the selective vulnerability of CA1 to tGCI stress^6,32,33^. Studies have also highlighted the role of several phospholipids and sphingolipids species in the mechanism of neuronal response after tGCI^6,31,34,35^, however, a comprehensive metabolomics approach to study the neuronal changes after tGCI is yet to be performed.

In this work, we benefited from the unique features of the tGCI model to try and answer several questions related to neuronal vulnerability to ischemia. First, we wanted to explore what metabolites and pathways would change significantly following tGCI in each CA1 and CA3 sub-regions, and how these enriched pathways could be related to the neuronal phenotype following tGCI using an untargeted metabolomics analysis approach. Second, we wanted to explore how early can we detect changes at the level of the metabolome following tGCI, and how can these changes be relevant to the subsequent apoptosis or survival in CA1 or CA3 respectively.

## Results

### tGCI induces cell death in CA1 sub-region only

**Figure 1:**
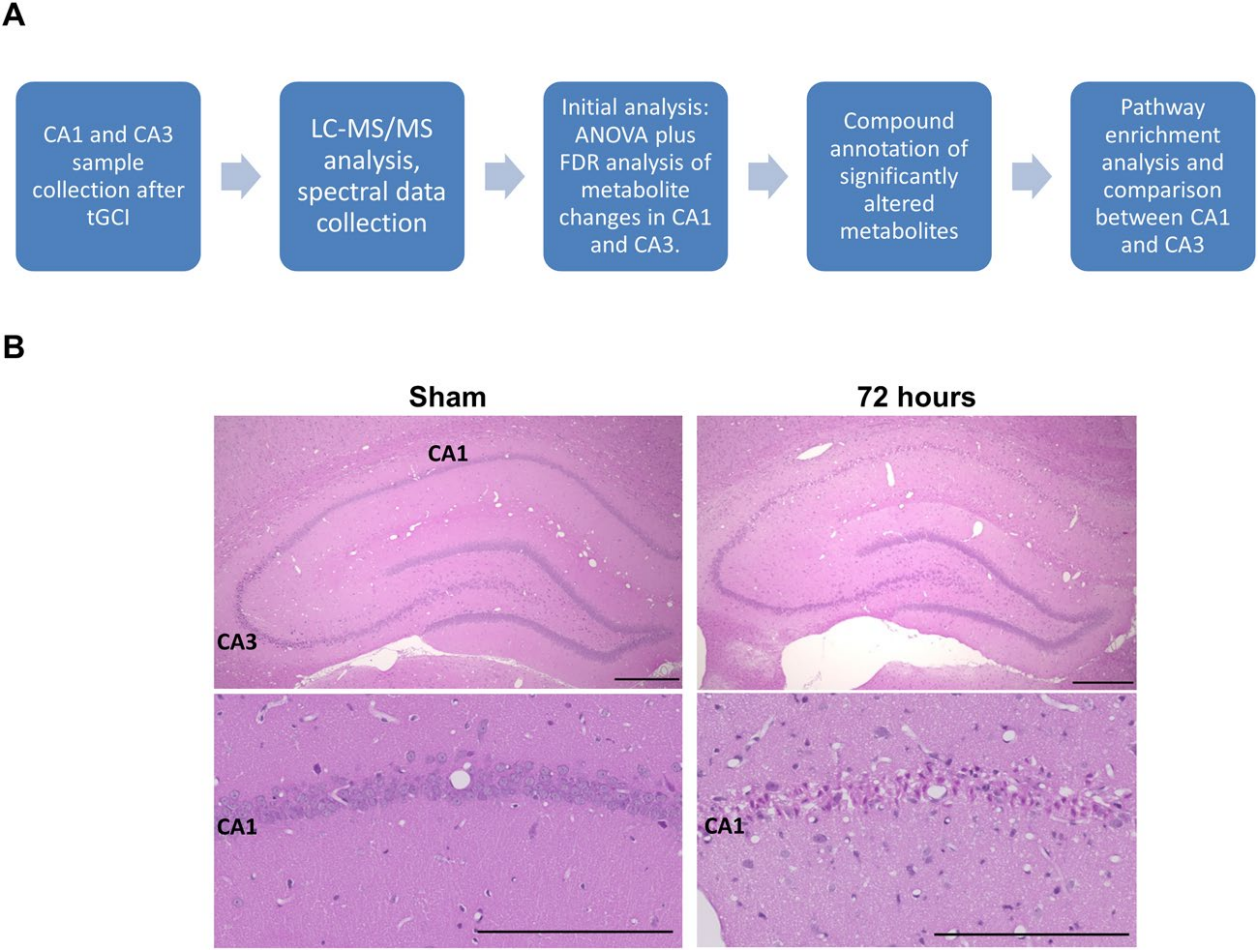
(**A**) Experimental workflow from collection of samples to pathway enrichment analysis. (**B**) H&E staining of rat sections [Upper row: lower magnification, Bar = 500 µm, lower row: higher magnification, Bar = 250 µm], Sham and 72 hours, showing the loss of neurons in the CA1 sub-region of the hippocampus after 72 hours of tGCI as compared to the intact Sham operated rats. In the higher magnification images, small pyknotic neurons are observed indicating neuronal apoptosis.

Histologic staining with Hematoxylin and Eosin showed that tGCI induced cell death in CA1 sub-region of the hippocampus 72 hours after tGCI, while the CA3 sub-region was largely unaffected as we previously reported^6,30^ [Fig. 1B]. After 72 hours of tGCI CA1 hippocampal neurons became small, pyknotic and dense, indicating that the neuronal damage is mainly due to apoptosis [Fig. 1B, Lower row].

### tGCI induced widespread metabolome changes in CA1 and CA3

**Figure 2:**
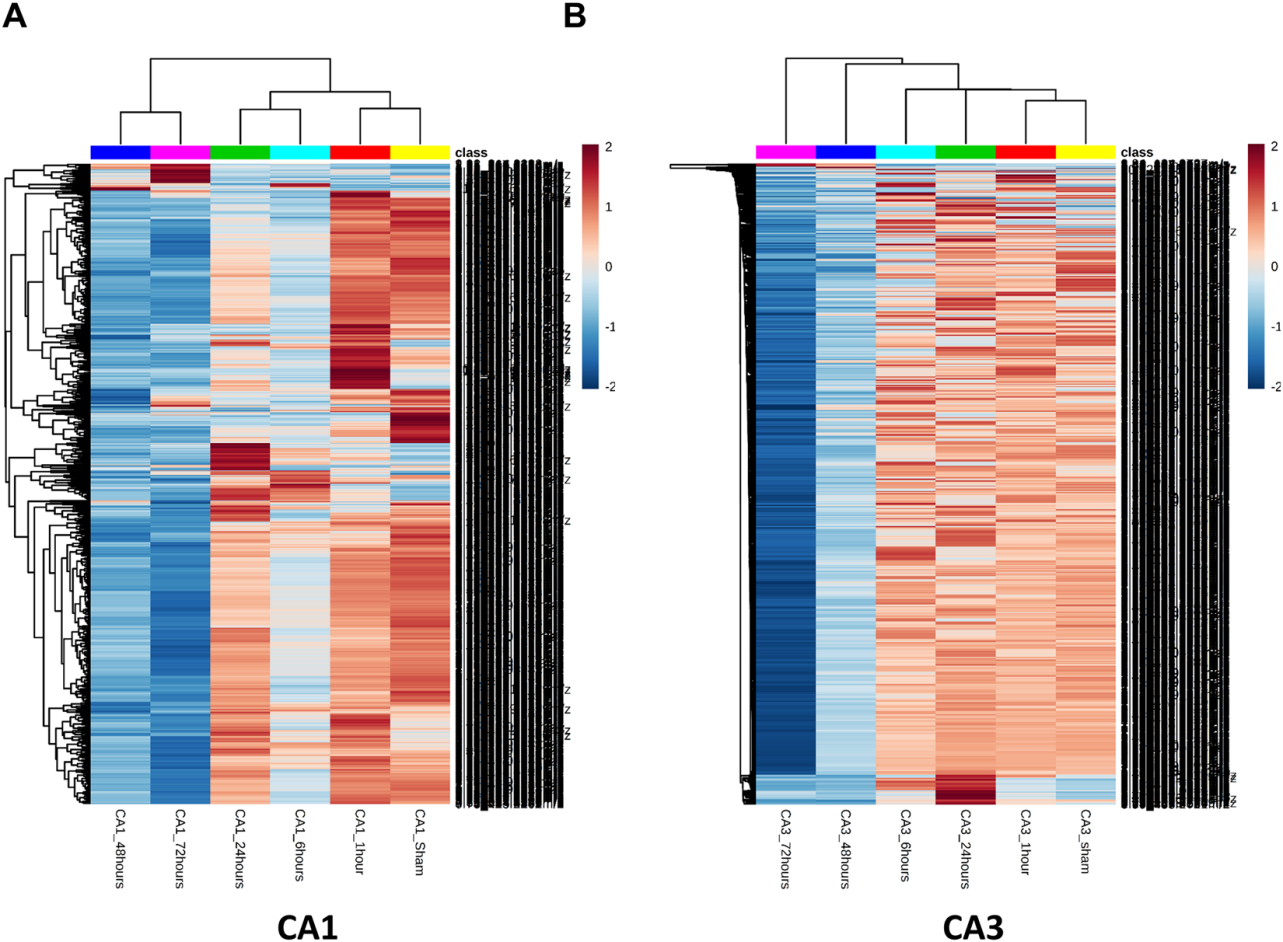
Heatmaps of un-annotated features showing changes in CA1 (**A)** and CA3 (**B**) in both negative and positive modes (top 1000 significant features on ANOVA). Significant clustering pattern can be observed in CA1 between the metabolite changes observed at 48 and 72 hours versus all other time points. Changes can also be observed in several metabolites between Sham and 6 hours. In CA3 the clustering is less sharply demarcated as in CA1, yet it is observable in the later time points. The lists of enriched compounds can be found in the Supplementary Data. Each time point is comprised of 5 animal samples (N = 5).

**Figure 3:**
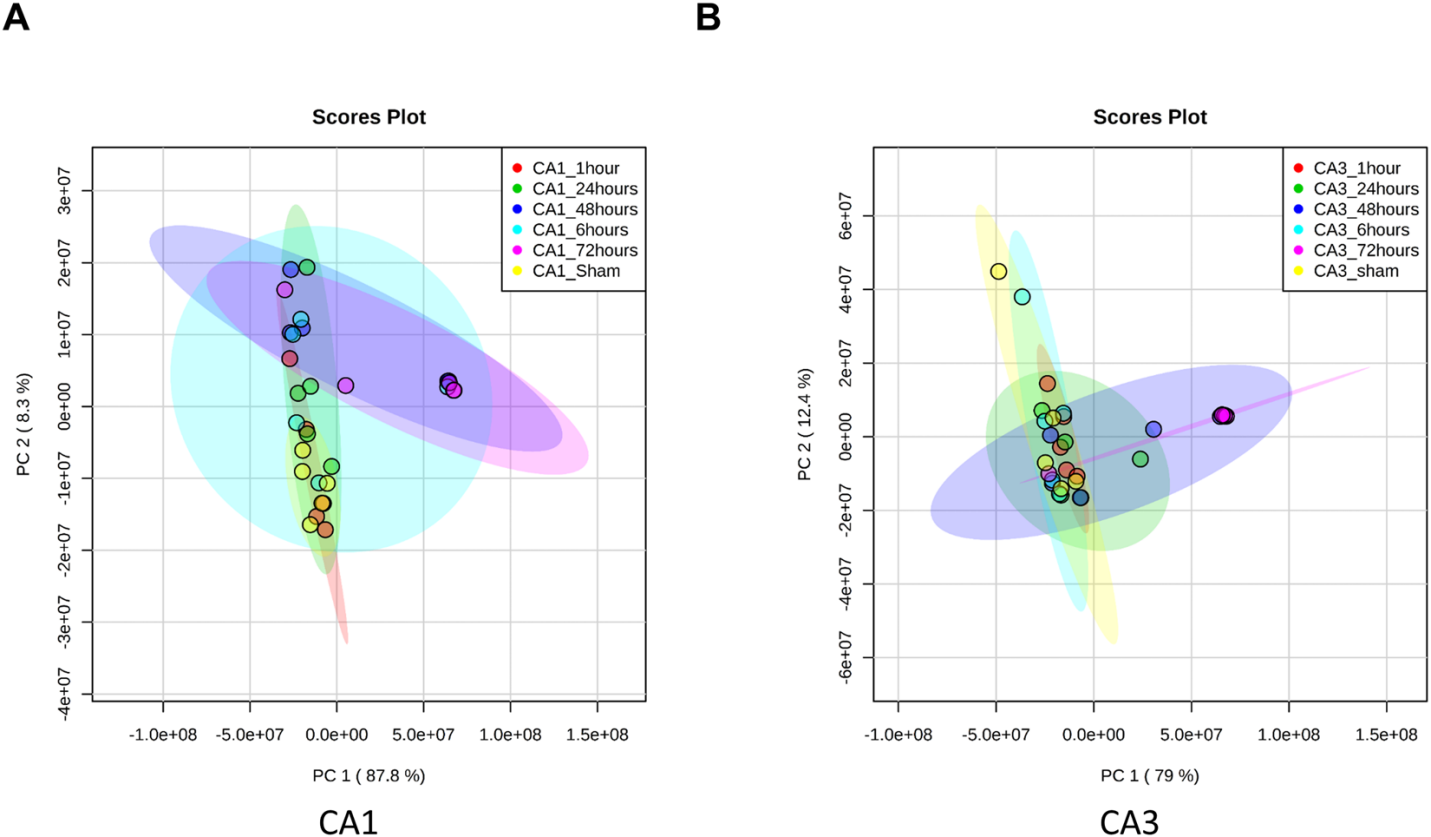
Principle component analysis for CA1 **(A)** and CA3 **(B)** confirming a more discrete and robust clustering pattern in CA1 that CA3.

LC-MS/MS analysis in both positive and negative modes was conducted on CA1 and CA3 samples at 1, 6, 24, 48 and 72 hours plus sham operated animals. In both regions there were significant changes in metabolites observed throughout the time course from initiation of global ischemia to apoptosis. In both region; metabolite changes showed clustering between 48 hours and 72 hours in a cluster, Sham and 1 hour in another cluster and 6 hour and 24 hours in a third cluster [Fig. 2]. The clustering was more discrete in the CA1 region compared to CA3. This clustering pattern was further confirmed using principle component analysis (PCA). Again, a more discrete and robust clustering pattern was observed in CA1 compared to CA3 [Fig. 3A,B].

**Figure 4:**
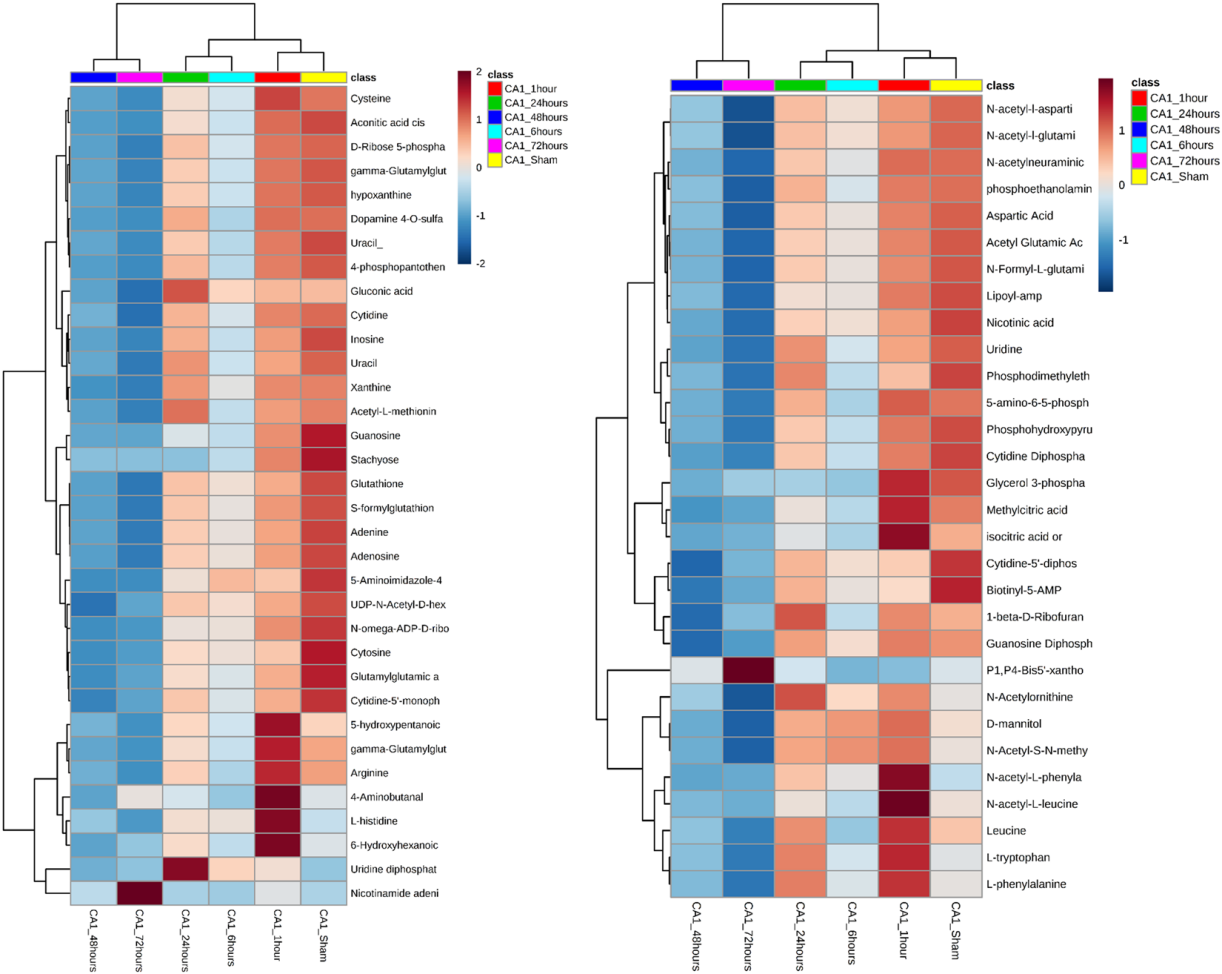
Heatmaps of statistically significant and successfully annotated compounds in CA1. Details of the metabolites are provided in Supplementary Table 1.

**Figure 5:**
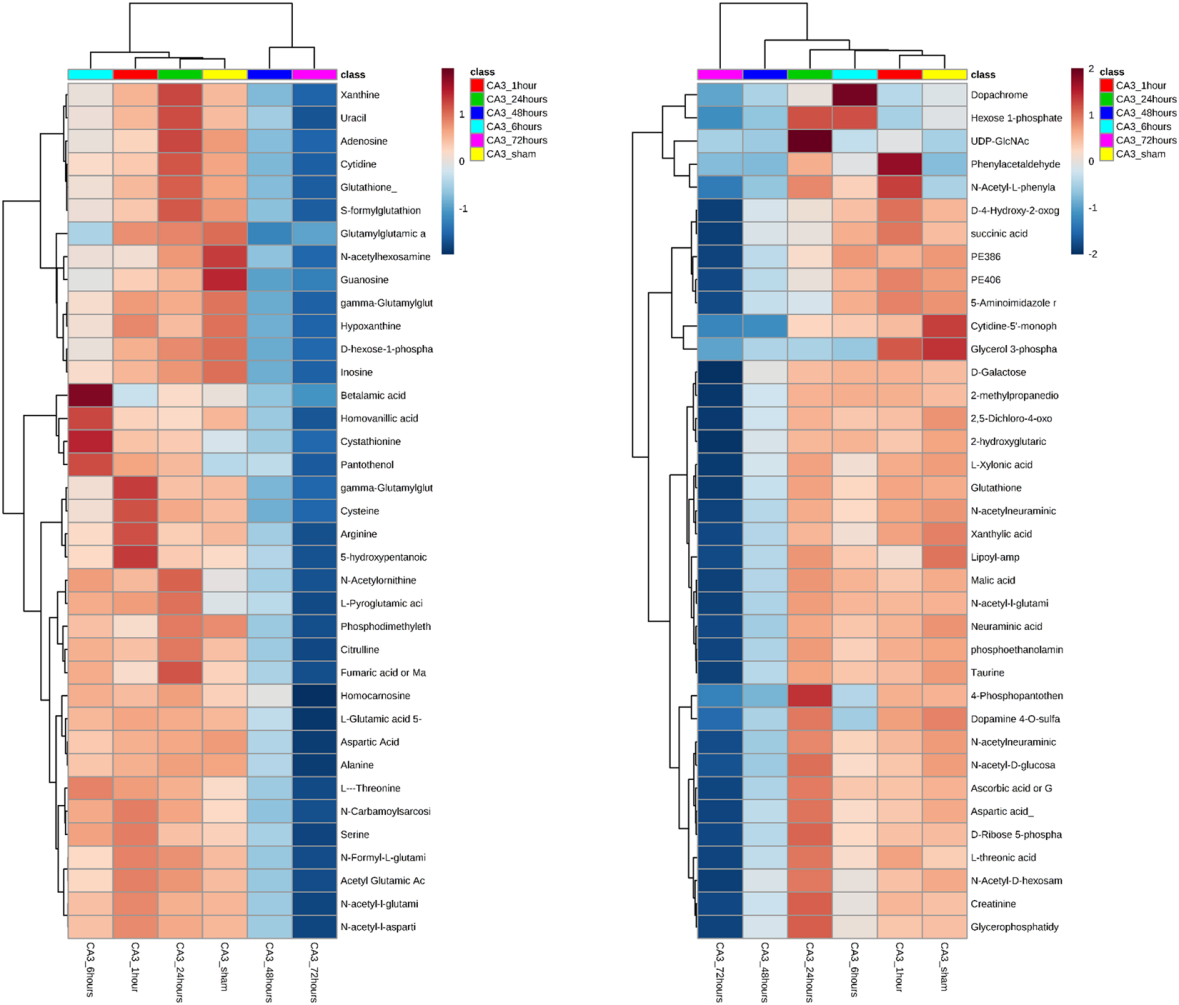
Heatmaps of statistically significant and successfully annotated compounds in CA3. Details of the metabolites are provided in Supplementary Table 2.

In the next step we performed statistical analysis in both CA1 and CA3 independently to highlight the significant metabolites. These metabolites were subjected to manual annotation and Fragmented compounds or unknown compounds were excluded from the downstream analysis. In the CA1 sub-region, there was a total of 384 significant features out of an initial pool of 3369 feature hits at the beginning of the analysis. Out of these 384 features, 64 compounds were successfully annotated [Fig. 4, Supplementary Table 1]. In the CA3 sub-region, there was a total of 834 significant features out of the initial pool of 3369 features. Of these compounds, 74 compounds were successfully annotated [Fig. 5, Supplementary Table 2]. Annotated compounds showed the same pattern of clustering observed when plotting the full dataset of compounds [Supplementary Figs. 1 and 2].

**Figure 6:**
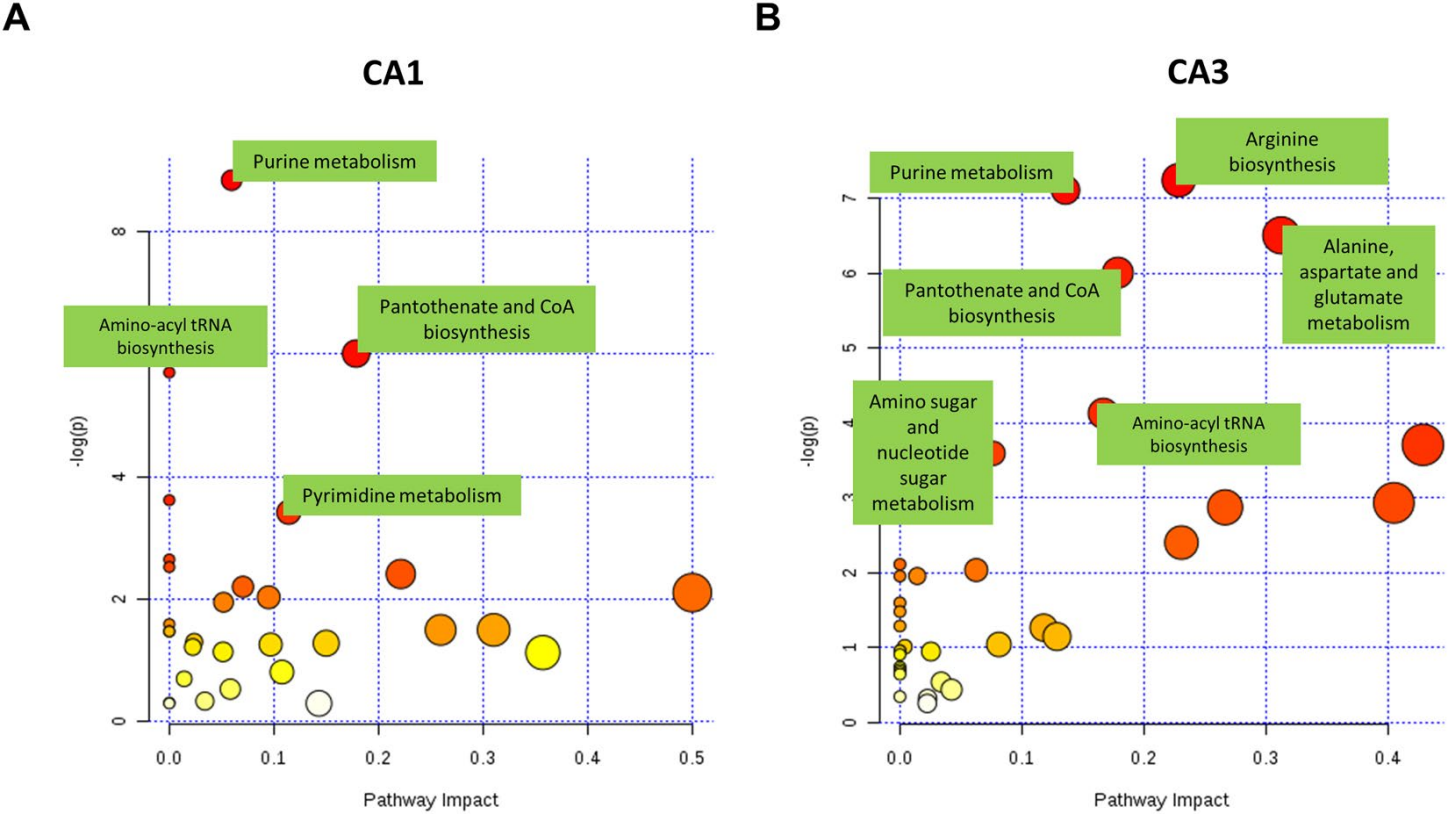
Pathway enrichment and topology analysis; **(A)** in CA1 sub-region and **(B)** in CA3 sub-region. Several pathways are enriched after tGCI, especially purine and pyrimidine metabolism, Pantothenate and CoA biosynthesis, Arginine biosynthesis and amino-acyl tRNA biosynthesis. The list of pathways is available in the Supplementary Tables 3 and 4.

Next, we analyzed the enriched metabolic pathways in both the CA1 and the CA3 sub-regions using the annotated metabolite sets from both regions using the pathway analysis feature which integrates enrichment analysis with pathway topology analysis^36^. Pathway analysis and topology integration showed that Pyrimidine metabolism, Purine metabolism, Pantothenate and CoA biosynthesis and Aminoacyl tRNA synthesis and beta-alanine metabolism were significantly enriched pathways with the highest impact in CA1 sub-region (*p* < 0.05) [Fig. 6A, Supplementary Table 3]. while Arginine biosynthesis, Alanine, aspartate and glutamate metabolism, Purine metabolism, Amino sugar and nucleotide sugar metabolism, Pantothenate and CoA biosynthesis and taurine and hypotaurine metabolism were the significant in CA3 sub-region (*p* < 0.05) [Fig. 6B, Table 1, Supplementary Table 4].

**Table 1:** Significantly enriched Pathways in CA1 and CA3.

CA1								
Pathway	Total	Expected	Hits	Raw p	−logP	Holm adjust	FDR	Impact
Purine metabolism	66	2.0994	9	0.000146	8.835	0.012227	0.012227	0.05958
Pantothenate and CoA biosynthesis	19	0.60437	4	0.00246	6.0074	0.20422	0.093769	0.17857
Aminoacyl-tRNA biosynthesis	48	1.5268	6	0.003349	5.6991	0.27461	0.093769	0
beta-Alanine metabolism	21	0.66799	3	0.026865	3.6169	1	0.5499	0
Pyrimidine metabolism	39	1.2406	4	0.032732	3.4194	1	0.5499	0.1142
**CA3**								
Arginine biosynthesis	14	0.44533	4	0.000715	7.2436	0.060039	0.034261	0.22843
Purine metabolism	66	2.0994	8	0.000816	7.1114	0.067706	0.034261	0.13571
Alanine, aspartate and glutamate metabolism	28	0.89066	5	0.001494	6.5066	0.12248	0.041821	0.3125
Pantothenate and CoA biosynthesis	19	0.60437	4	0.00246	6.0074	0.1993	0.051669	0.17857
Aminoacyl-tRNA biosynthesis	48	1.5268	5	0.016121	4.1276	1	0.27083	0.16667
Taurine and hypotaurine metabolism	8	0.25447	2	0.024554	3.7069	1	0.33038	0.42857
Amino sugar and nucleotide sugar metabolism	37	1.1769	4	0.027531	3.5924	1	0.33038	0.07631

### Analysis of differential pathway enrichment between CA1 and CA3

Since PCA showed that CA1 and CA3 sub-regions’ metabolome profile to be more divergent at early time points, we attempted to evaluate this notion in light of the top enriched pathways in each region. We selected the statistically significantly enriched pathways, based on the MetaboAnalyst pathway analysis function, and identified compounds pertaining to each pathway in our dataset. Next, we compared these compounds at each time point starting from 1 hour to before the onset of apoptosis (i.e. 48 hours) between the 2 sub-regions using Student’s t test. Overall, there were 9 significantly enriched pathways in CA1 and CA3. The analysis of metabolites related to each pathway revealed that the most significant differences were at the earlier time points of 1 and 6 hours. While minimal to no changes were observed in later time points. Purine metabolism, pyrimidine metabolism, amino sugar and nucleotide sugar metabolism, arginine biosynthesis and alanine, aspartate and glutamate metabolism pathways showed significant differences between CA1 and CA3 in some of the metabolites related to these pathways. The list of metabolites and details statistical analysis can be found in Supplementary Table 5.

### **Metabolomics reveal very early changes after tGCI**

In this step, we decided to explore the early changes in metabolomic profiles of both CA1 and CA3 sub-regions that can be detected prior to the overt apoptosis phenotype observation beyond the metabolites belonging to particular enriched pathways and with more stringent parameters. We performed Student’s t-test statistical analysis-based comparison between CA1 and CA3 one hour after tGCI with all the compounds in our dataset. Compounds with fold change >2 and *p* < 0.05 were considered significant.

One hour after tGCI, 12 compounds were significantly differentially enriched (Fold change >2, *p* < 0.05) in CA1 vs CA3, with 6 of these compounds upregulated and 6 downregulated in CA1 vs CA3 [Table 2].

**Table 2:** Differentially enriched and significant metabolites at each time point following tGCI between CA1 and CA3.

Compound	*t*R_*m/z*	Detected	Formula	Neutral	Adduct	Theoritical m/z	Delta ppm	Status	Mode	PubChem ID	Mean (SD) of CA1_1hour	Mean (SD) of CA3_1hour	p-value	q-value (FDR)	Fold Change CA1/CA3
**CA1 vs CA3 after 1 hour of tGCI**															
Guanosine	4.17_282.0836 m/z	282.0836	C10H13N5O5	283.0917	M-H	282.0844	2.8	C	Negative	6802	27261.734 (12973.578)	8599.647 (3794.538)	0.0297	0.1708	3.17
Adenosine diphosphate ribose	5.04_558.0632 m/z	558.0632	C15H23N5O14P2	559.0717	M-H	558.0644	2.2	P	Negative	11988267	126701.269 (51908.538)	52407.297 (43361.399)	0.0396	0.186	2.42
Deoxyadenosine	1.54_274.0926 m/z	274.0926	C10H13N5O3	251.1018	M + Na	274.0911	−5.5	P	Positive	13730 or 439182	380.422 (180.680)	166.099 (86.692)	0.0438	0.5932	2.29
UDP-hexose	5.28_565.0467 m/z	565.0467	C15H24N2O17P2	566.055	M-H	565.0477	1.8	P	Negative	8629	119599.859 (50445.561)	55673.996 (30356.594)	0.0413	0.188	2.15
Nicotinamide adenine dinucleotide (NAD + )	2.95_664.1144 m/z	664.1144	C21H28N7O14P2	664.1169	M + H	664.1169	3.8	C	Positive	5892	1018.984 (298.928)	495.805 (266.467)	0.0193	0.5932	2.06
GDP-hexose	5.58_604.0691 m/z	604.0691	C16H25N5O16P2	605.0772	M-H	604.0699	1.3	P	Negative	644106	11726.265 (4704.345)	5822.306 (2562.261)	0.039	0.186	2.01
Dehydroascorbic Acid	1.49_173.0086 m/z	173.0086	C6H6O6	174.0164	M-H	173.0092	3.5	P	Negative	440667	215710.361 (50313.917)	448686.831 (153634.118)	0.0122	0.1559	−2.08
2-Methylcitric acid	1.49_205.0348 m/z	205.0348	C7H10O7	206.0427	M-H	205.0354	2.9	P	Negative	515	161322.192 (35502.350)	342757.941 (121684.961)	0.0264	0.1708	−2.12
PI(38:4)	2.62_885.5477 m/z	885.5477	C47H83O13P	886.5571	M-H	885.5499	2.5	P	Negative	71581166	8606071.917 (4557321.866)	18321615.444 (7251849.721)	0.0349	0.1773	−2.13
cis-Acetylacrylate	2.18_97.0281 m/z	97.0281	C5H6O3	114.0317	M + H-H2O	97.029	9.3	P	Positive	5281015	43.152 (12.371)	94.676 (32.508)	0.0107	0.5932	−2.19
Ribose 1,5-bisphosphate	3.72_308.9834 m/z	308.9834	C5H12O11P2	309.9855	M-H	308.9782	−16.8	P	Negative	14035695	5271.256 (3512.565)	12410.674 (4728.477)	0.0266	0.1708	−2.35
Hexose 1-phosphate	4.52_241.0115 m/z	241.0115	C6H13O9P	260.0297	M-H20-H	241.0113	−0.8	P	Negative	123912	1734.493 (1187.248)	5401.668 (1437.128)	0.0023	0.1227	−3.11
**CA1 vs CA3 after 6 hours of tGCI**															
20-Hydroxy-leukotriene E4	4.66_494.2011m/z	494.2011	C23H37NO6S	455.2342	M + K	494.1973	−7.7	P	Positive	5280606	24.728 (12.515)	9.881 (7.549)	0.0528	0.6772	2.5
4-Amino-5-imidazole carboxamide	4.12_109.0510 m/z	109.0510	C4H6N4O	126.0542	M + H-H2O	109.0515	4.6	P	Positive	9679	71.731 (20.892)	30.899 (8.235)	0.0036	0.6772	2.32
N1-Acetylspermidine	0.82_188.1762m/z	188.1762	C9H21N3O	187.1685	M + H	188.1757	−2.7	P	Positive	496	191.185 (64.335)	84.299 (42.357)	0.0146	0.6772	2.27
Oxalosuccinic Acid	3.59_189.0038 m/z	189.0038	C6H6O7	190.0114	M-H	189.0041	1.6	P	Negative	972	557.773 (392.596)	1157.188 (198.478)	0.0159	0.4383	−2.07
Histidinyl-Phenylalanine	1.40_283.1180 m/z	283.1180	C15H18N4O3	302.1379	M-H20-H	283.1195	5.3	P	Negative	4466133	6195.458 (4086.461)	13075.953 (4140.117)	0.0295	0.5589	−2.11
PE(P-36:4)	1.37_722.5114 m/z	722.5114	C41H74NO7P	723.5203	M-H	722.513	2.2	P	Negative	52925126	406885.955 (245192.024)	865995.714 (22915.326)	0.0135	0.4383	−2.13
**CA1 vs CA3 after 72 hours of tGCI**															
4-Amino-5-imidazole carboxamide	4.12_109.0510 m/z	109.0510	C4H6N4O	126.0542	M + H-H2O	109.0515	4.6	P	Positive	9679	24.655 (10.269)	11.357 (7.168)	0.0323	0.7441	2.17
Deoxyuridine triphosphate	2.03_468.9770 m/z	468.9770	C9H15N2O14P3	467.9736	M + H	468.9809	8.3	P	Positive	65070	70.473 (52.528)	144.872 (43.218)	0.0296	0.7441	−2.06

**Pos:** positive mode. **Neg:** negative mode.

### Time point comparison between CA1 and CA3 reveals distinct metabolic signatures

Next, we performed statistical comparison between CA1 and CA3 at each time point until the onset of apoptosis. Using the same cut-off statistical values as before, we found out that after 6 hours of tGCI induction, 3 metabolites were upregulated in CA1 vs CA3, and 3 were downregulated [Table 2]. After 24 and 48 hours of tGCI we did not observe statistically significant metabolite differences between CA1 and CA3 based on our cut-off values. Some metabolites showed trends of increase and decrease with significant *p* < 0.05 but did not satisfy the cut-off value of fold change = 2 [Data not shown].

72 hours after tGCI, the time point at which apoptosis sets in our model and also microglia are recruited to CA1 subregion, as we previously demonstrated^6,30^; comparing CA1 and CA3 showed distinctly 1 metabolite to be upregulated in CA1 vs CA3, namely: 4-Amino-5-imidazole carboxamide; and 1 metabolite to be downregulated: Deoxyuridine-5’-triphosphate, both of which had a fold change increase of >2 and *p* < 0.05 [Table 2].

### PCA analysis for time point comparison

To confirm our observation that most of the changes after tGCI occur early, PCA analysis for each time point showed that at 1 and 6 hours, CA1 and CA3 clustered significantly opposite to each other, signifying differential metabolic profile in these two regions [Supplementary Figs. 1 and 2]. At 24 and 48 hours, the differences were not as clear, with samples from both regions overlapping, indicating less demarcation at the metabolome level [Supplementary Figs. 3 and 4]. At 72 hours, the differences became more apparent once again, especially in the positive mode, however not as sharp as the earlier time points [Supplementary Fig. 5]. These data confirm the patterns and trends observed with the statistical analysis.

## Discussion

Transient global cerebral ischemia is known to induce selective sub-acute neuronal apoptotic cell death in the CA1 sub-region of the hippocampus, with an interesting contrasting pattern of neuronal survival in the neighboring CA3 sub-region^6,30,33,37^. This contrast allows the study of factors that may contribute to neuronal vulnerability to ischemia, especially the subacute apoptotic pattern that allows the analysis of events prior to overt neuronal apoptosis^6^. The neuronal apoptosis in the CA1 sub-region follows the intrinsic pathway, with the release of Cytochrome *c* from the mitochondria, following mitochondrial outer membrane depolarization, as the initiating event of downstream apoptotic cascade^33^. Several mechanisms were reported to contribute to the selective vulnerability of the CA1 sub-region to tGCI, including; mitochondrial damage^38^, insufficient proteasome activity^32^, anomalies in Ca^2+^ homeostasis^39^, continuous neuronal agitation^40^ and phospholipid metabolic changes^31,35^. In a previous report we used LC-MS/MS to evaluate sphingosine-1-phosphate (S1P) metabolism, combined by gene analysis of members of the cascade of S1P synthesis, degradation and export^6^. In that report, we observed an early upregulation of S1P in CA3 that was not observed in CA1, and we hypothesized that this intracellular S1P upregulation is one of the mechanisms by which the CA3 sub-region evades cell death after tGCI. Previous studies using global cerebral ischemia model have observed several metabolic changes to occur in the hippocampus, such as changes in phosphatidylcholine^31^ and other phospholipids^35^ as well as small metabolites and amino acids^41^, however, none of these studies attempted an approach as wide-scoped as the one reported here.

In our work, we highlighted several pathways that were enriched following transient global cerebral ischemia in both CA1 and CA3 sub-regions. In the CA1 sub-region, several purine and pyrimidine molecules were differentially expression early after tGCI compared to CA3, and purine and pyrimidine pathways were amongst the highest enriched pathways in this area following tGCI. Purine and pyrimidine metabolism perturbations may lead to, or reflect, defective DNA synthesis or repair, further shifting the neurons towards apoptosis^42,43^. Amino sugar and nucleotide sugar metabolic pathway, which was enriched in CA3 subregion, also showed an interesting trend following tGCI. This pathway is involved in glycosylation protein modifications, which is the most abundant post-translational protein modification, in the Golgi apparatus^44,45^ and is related to host-pathogen responses in plants and mammals^46,47^. Nucleotide sugars are important for the glycosylation of proteins, lipids and proteoglycans, and perturbation in these processes can lead to defects in development, organogenesis and immunity^46^. Nonetheless, we could not find a causal relationship between perturbations in this pathway and neuronal apoptosis in the literature, however, it will be interesting to study it in depth as it may be related to cell stress pathways during neuronal apoptosis.

We also wanted to identify metabolites that can be linked to apoptosis or resistance to ischemia in each topographic region and can also be linked to early or delayed events leading to apoptosis. Interestingly, when we used all our dataset and compared between CA1 and CA3 subregions at each time point studied, we observed the most significant metabolite changes to occur very early after tGCI, prior to any phenotypically observable changes. Notably, the metabolites upregulated in CA1 after 1 hour of tGCI included several nucleotides such as Adenosine diphosphate ribose, Deoxyadenosine and Guanosine, which are purine metabolites, and are linked to ribonucleic acid synthesis, and their upregulation maybe be linked to increased transcription in the CA1 subregion to cope with the oxidative stress induced by tGCI. Interestingly, in a previous report^6^ we observed that HSP-70, one of the main cytosolic chaperones and a heat shock response and unfolded protein response regulator^48^, was several hundred folds upregulated in CA1 vs CA3 after 6 hours of tGCI. Thus, we can from these 2 observations, plus the aforementioned upregulation of several nucleotide sugars, hypothesize that the upregulation of HSP-70 observed previously to possibly be a response to an increased and aberrant transcription and translation occurring in the CA1 after tGCI, in order to correct the resulting disruption of the protein homeostasis. Notwithstanding, the exact cause of this hypothesized mechanism is not clear at the moment, but it sure warrants additional research.

After 72 hours of tGCI, we consistently observed neuronal apoptosis in the CA1 sub-region only, and recruitment of microglia to clear the small pyknotic dead neurons from this regions was reported previously at the same time point^6^. We compared CA1 and CA3 subregions’ metabolite signature at this time point to evaluate changes pertaining to such a process. In the CA1, there were 1 upregulated and 1 downregulated metabolite compared to the CA3. 4-Amino-5-imidazole carboxamide, a purine metabolite upregulated in the CA1 after 72 hours of tGCI, was reported to have apoptotic effects^49,50^, therefore its decrease in the CA3 compared to the CA1 may represent a mechanism by which the CA3 evades apoptosis. However, Deoxyuridine-5’-triphosphate, downregulated in CA1 vs CA3, is another metabolite which accumulation is linked to a commitment to apoptosis and cell growth retardation^51^. These seemingly contradictory expression of the 2 metabolites might be due the fact that 72 hours after tGCI microglia are recruited to clear out the apoptotic neurons^6^. Therefore, the metabolic pattern maybe a reflection of microglial activity and not only neuronal apoptosis.

Indeed, certain inherent limitations of this work should be considered. We employed an untargeted metabolomics approach. While we followed a very stringent protocol for identification and statistical analysis, errors in annotation (which is performed putatively and manually in some compounds) are a possibility. While we do not believe these errors will affect the results at large (such as the pathway analysis for example), we recommend that any follow-up work building on these findings to confirm the results of the target metabolite using a targeted metabolomics approach as a first step. Additionally, the presented metabolic profiling was limited by following a methodology regarding extraction and detection of non-polar and neutral metabolites because of the simple process of deproteinization using methanol. This method should be improved to cover a wider range of metabolites in future studies.

In summary, we offer a new metabolomics-based insight into the basis of selective vulnerability of a subset of hippocampal neurons to global cerebral ischemia, by employing metabolomic profiling tools. Through this approach, we were able to identify several enriched pathways, not reported previously in this context, that can be linked to selective neuronal vulnerability or resistance to ischemia, which can be further explored and possibly exploited for targeted therapy in cerebral ischemia. Moreover, we showed that metabolomic analysis can capture very early changes pertaining to neuronal apoptosis, well before any phenotypical evidence of such apoptosis. Strikingly, we observed most significant metabolic changes to occur very early after tGCI, contrary to our expectations, indicating that very early metabolic changes dictate neuronal cell fate after ischemia. This finding should be considered in future metabolomic work aiming to assess neuronal cell death and also the death of non-neuronal cell types.

## Methods

### tGCI model

tGCI model was performed by bilateral common carotid artery occlusion coupled with severe hypotension as described previously^6,30^. Seven weeks old male Sprague–Dawley rats weighing 280–320 g were used in this study. Animals were bought from a local vendor (Kumagai-Shoten Sendai) and housed in a controlled environment with 12 hours light/dark cycle and temperature regulated at 23 °C. Animals were housed in clear plastic cages with free access to water and food. A maximum of 4 animals were housed in a single cage. Animals were allowed to acclimatize for at least 48 hours before any experimental procedures. All experiments were conducted according to protocols approved by the animal care facility of Tohoku University (Ethical approval number 2017MdA-137–3) and according to the ARRIVE (Animal Research: Reporting *In Vivo* Experiments) guidelines. All animal surgeries were performed between 9am and 4 pm. No blinding was performed for this work.

### Surgery

Briefly; the animals were anesthetized by a gas mixture of 70% nitrous oxide and 30% oxygen containing 1.5% isoflurane. The femoral artery was cannulated and connected to a blood pressure monitor, and blood was then aspirated through the external jugular vein until the blood pressure dropped to around 25 mmHg. Both common carotid arteries were occluded for 5 min using aneurysm clips, while maintaining the blood pressure in the range of 25–30 mmHg (mean: 28 mmHg). Animals were allowed to recover slowly after surgery undisturbed. tGCI does not induce any overt motor deficits or long-lasting distress in the animals. No pain medications were given after the surgery for pain control in order not to interfere with the metabolomics analysis.

Following reperfusion, the animals were sacrificed at the designated time points (1 hour, 6 hours, 24 hours, 48 hours and 72 hours). Sham control animals were prepared using the same procedure, omitting the ischemia/hypoperfusion step and were sacrificed after 72 h. A total of 60 animals were used for this study, 10 at each time point. All animals were included in the study. There were no predetermined exclusion criteria. No animals died after tGCI or during the surgery.

Animals were randomized based on a simple randomization scheme. Each animal was randomly assigned a number between 1 to 60, and the random number generator function of Excel (Office 365, Microsoft) [=RANDBETWEEN(1, 60)] was used to match this number with a time-group which was arranged chronologically in the next column and to an experimental group (histology versus mass spectrometry).

### Histology

Animals were sacrificed after 1, 6, 24, 48 and 72 hours of tGCI plus sham operated animals (N = 5 at each time point, total 30), by overdose inhalation of isoflurane and transcardiac perfusion with ice-cold saline followed by transcardiac perfusion of 4% paraformaldehyde (PFA) in 0.1 M phosphate-buffered saline (PBS; pH 7.4) [Nacalai tesque. Catalog # 09154–85]. Brains were dissected, cut on a matrix, and stored in 4 °C in 4% paraformaldehyde. Brains were prepared as paraffin blocks. Paraffin blocks were cut at a thickness of 5 µm, de-paraffinized and stained with Hematoxylin and Eosin (H&E) stain. Sections were observed and photographed using a multi-channel microscope (BZ-9000, Leica. RRID:SCR_015486).

### Tissue preparation procedure for LC-MS/MS

A total of 30 animals were used for metabolomics analysis (N = 5 per time-point). Animals were sacrificed after 1, 6, 24, 48 and 72 hours of tGCI, plus sham operated animals, by overdose inhalation of isoflurane and transcardiac perfusion with ice-cold saline. The brains were extracted and sliced into 2 mm sections on a matrix and the CA1 and CA3 sub-regions dissected under microscope on a glass plate placed on ice. Collected tissues were flash frozen in liquid nitrogen, weighed and stored at −80 °C until the time of a high-performance liquid chromatography Fourier transform mass spectrometry (LC-FTMS) and an ultra-high-performance liquid chromatography quadrupole time-of-flight mass spectrometry (UHPLC-QTOF/MS) analysis. Between 0.02 and 0.045 grams were collected per sample. Methanol containing 0.1% formic acid (200 µL) was added to the samples. After homogenization with beads at 5,500 rpm for 20 sec (2 pieces of 2.8 mm zirconium oxide beads) and sonication in an ultrasonic bath for 10 min, the samples were centrifuged at 16,400 × g for 20 min at 4 °C. Then, the supernatant (120 µL) was transferred to a 96-well plate (700 µL round well; Waters Corp., Milford, MA) and diluted (x2) with water containing 0.1% formic acid. The preparation of study quality control (SQC) and dilution quality control (dQC) and the run order of samples for G-Met analysis were performed as described in a previous report^52^.

### LC-FTMS and UHPLC-QTOF/MS analyses

Tandem Mass spectrometry was performed as previously described^52^. The UHPLC-QTOF/MS analysis was performed on an Acquity Ultra Performance LC I-class system, equipped with a binary solvent manager, a sample manager, and a column heater (Waters Corp.). This system interfaced with a Waters Synapt G2-Si QTOF MS with electrospray ionization (ESI) system, operated in positive mode. LC separation was performed using a C18 column (Acquity HSS T3; 150 mm × 2.1 mm i.d., 1.8 µm particle size; Waters, Catalog # Part No.186003540) with a gradient elution of solvent A (water containing 0.01% formic acid (Wako, Japan. Catalog # 067–04531) and solvent B (acetonitrile (Kanto Kagaku, Japan, Catalog # 01031–2B) containing 0.01% formic acid) at 400 µL/min. The data were collected using MassLynx, v4.1 software (Waters Corp., Manchester, UK. RRID:SCR_014271).

The LC-FTMS system consisted of a NANOSPACE SI-II HPLC, equipped with a dual pump system, an auto sampler, and a column oven (Shiseido, Tokyo, Japan), and a Q Exactive Orbitrap MS (Thermo Fisher Scientific, San Jose, CA) equipped with a heated-ESI-II (HESI-II) source for negative ion mode analysis. LC separation was performed using a HILIC column (ZIC-pHILIC; 100 mm × 2.1 mm i.d., 5 µm particle size; Sequant, Darmstadt, Germany. Catalog # 1.50462.0001) with a gradient elution of solvent A (10 mmol L − 1 ammonium bicarbonate (Cell Science & Technology Inst.,Inc. Catalog # cn2104a0) in water, pH 9.2) and solvent B (acetonitrile (Kanto Kagaku, Japan, Catalog # 01031–2B)) at 300 µL/min. The data were collected using Xcalibur v2.2 software (Thermo Fisher Scientific, San Jose, CA. RRID:SCR_014593). More details can be found in previous reports^52^.

### Data analysis and statistics and compound annotation

The data analysis was performed as previously reported^52^. Features were selected based on their coefficient of variation (CV) with the SQC samples, which were injected after every 8 study samples; features with CV over 30% were eliminated. Features were also positively selected according to the inverse correlation of the dilution fold and the peak intensity to the dQC samples, as well as their CV with 3 injections of the same dQC samples. The metabolomics data in negative ion mode by LC-FTMS and positive ion mode by UHPLC-QTOF/MS were processed using the Xcalibur software (version 2.2, Thermo Fisher Scientific) and MassLynx software (version 4.1, Waters) respectively. RAW files were loaded into the Progenesis QI software (Nonlinear Dynamics, Newcastle, UK), aligned using automatic alignment and the samples were grouped according to experimental conditions (CA1 or CA3 - Time point of collection). Data normalization was performed according to sample weight and each feature was represented by unit per microgram of brain tissue prior to statistical analysis.

### Statistical analysis

First, using the statistical analysis feature of the MetaboAnalyst software v4.0^36^, ANOVA testing with Fisher’s post hoc analysis plus false discovery rate (FDR) analysis were performed on the datasets obtained from CA1 and CA3 across different time points (each area tested separately). Features with more than 50% missing values were excluded from the statistical analysis (when a given feature was absent (i.e. not detected) from 50% or more of the samples in one group this feature was excluded from downstream analysis). Significant features were considered to have *p* < 0.05 on ANOVA and FDR (q) <0.05. For time point comparison between CA1 and CA3, Student’s t test was performed. Features with *p* < 0.05 plus fold change of >2 were considered significant. Next, the significant features were imported into SPSS v.20 (IBM corp.) and multivariate analysis with ANOVA and Fisher’s post hoc plus Levene test (test for homogeneity of variance) was conducted and metabolites with a value of > 0.05 on Levene test were excluded from further downstream analysis and only features that scored <0.05 on Levene test were identified and analyzed. For time point comparison between CA1 and CA3, student’s t-test was conducted using MetaboAnalyst software, and metabolites with *p* < 0.05 and fold change ≥2 were considered significant.

### Principal component analysis (PCA) and power analysis

Principle component analysis was performed using the Progenesis QI software and Ezinfo software (version 3.0.3, Waters). Power analysis was performed using the power analysis function of the MetaboAnalyst software^36^ using the whole dataset as an input.

Using our dataset of feature as an input for power analysis yielded an unrealistic estimate of more than 200 samples per time point in order to achieve a power of ≥0.8 (data not shown). Since this number is logistically implausible, plus given our aim of conducting an explorative untargeted assay, we evaluated the results of principle component analysis to show weather significant and robust differences exist in our data or not.

### Extracted features and identified compounds

Features that were found to be significant with statistical analysis were manually annotated using the Progenesis QI database using their MS/MS peak data and fragmented features without MS/MS peak data or features that could not be assigned an annotation were excluded from further downstream analysis. Features were assigned one or more annotations based on their scores and similarities. Features with more than one annotation were assigned a single annotation in downstream analysis if all annotations belonged to the same class or pathway, or all the annotations were used in enrichment analysis if less than three annotations were assigned and belonging to different classes. No feature was assigned more than three annotations. The annotated features were further confirmed using chemical standards or other databases as the Jmorp database (https://jmorp.megabank.tohoku.ac.jp/201808), Human Metabolome Database (http://www.hmdb.ca, RRID:SCR_007712), PubChem database (https://pubchem.ncbi.nlm.nih.gov. RRID:SCR_004284) and Chemspider (http://www.chemspider.com, RRID:SCR_006360) when chemical standards were not available.

### Pathway and enrichment analysis

We used the pathway analysis and topology functions of MetaboAnalyst software to analyze which pathways were enriched in each hippocampal sub-region. Significant pathways were analyzed for their enriched metabolites in both CA1 and CA3 sub-regions. These metabolites were compared using Student’s T test at each time point to show significant differences in enrichment in both hippocampal regions. Metabolites with *p* < 0.05 and fold change ≥2 were considered significant.

## Supplementary information

Supplementary data.

## Data Availability

Raw unprocessed data were deposited at Metabolites (https://www.ebi.ac.uk/metabolights/index) study identifier: MTBLS1580. Additional information on the data can be requested from the corresponding authors.

## References

[1] Alonso A, Marsal S, Julia A (2015). Analytical methods in untargeted metabolomics: state of the art in 2015. Frontiers in Bioengineering and Biotechnology.

[2] Ferrario M (2016). Mortality prediction in patients with severe septic shock: a pilot study using a target metabolomics approach. Scientific Reports.

[3] Locasale JW (2012). Metabolomics of human cerebrospinal fluid identifies signatures of malignant glioma. Mol Cell Proteomics.

[4] Gao X (2017). Large-scale Metabolomic Analysis Reveals Potential Biomarkers for Early Stage Coronary Atherosclerosis. Scientific Reports.

[5] Dang, V. T., Huang, A. & Werstuck, G. H. Untargeted metabolomics in the discovery of novel biomarkers and therapeutic targets for atherosclerotic cardiovascular diseases. *Cardiovasc Hematol Disord Drug Targets*, 10.2174/1871529X18666180420170108 (2018). 10.2174/1871529X18666180420170108 29683098

[6] Rashad S (2018). Intracellular S1P Levels Dictate Fate of Different Regions of the Hippocampus following Transient Global Cerebral Ischemia. Neuroscience.

[7] Ivanisevic J (2016). Metabolic drift in the aging brain. Aging (Albany NY).

[8] Kopp F (2010). The glycerophospho metabolome and its influence on amino acid homeostasis revealed by brain metabolomics of GDE1(-/-) mice. Chem. Biol..

[9] Kumar KK (2015). Untargeted metabolic profiling identifies interactions between Huntington’s disease and neuronal manganese status. Metallomics.

[10] Parry, T. L. *et al*. Untargeted metabolomics analysis of ischemia-reperfusion-injured hearts *ex vivo* from sedentary and exercise-trained rats. *Metabolomics* **14**, 10.1007/s11306-017-1303-y (2018). 10.1007/s11306-017-1303-y PMC6086497 30104954

[11] Testai FD (2015). Changes in the metabolism of sphingolipids after subarachnoid hemorrhage. Journal of Neuroscience Research.

[12] Dettmer K, Aronov PA, Hammock BD (2007). Mass spectrometry-based metabolomics. Mass Spectrometry Reviews.

[13] Misra BB (2018). New tools and resources in metabolomics: 2016-2017. Electrophoresis.

[14] Ivanisevic J, Siuzdak G (2015). The Role of Metabolomics in Brain Metabolism Research. Journal of neuroimmune pharmacology: the official journal of the Society on NeuroImmune Pharmacology.

[15] Abe T (2018). Metabolomic Analysis of Mouse Brain after a Transient Middle Cerebral Artery Occlusion by Mass Spectrometry Imaging. Neurol. Med. Chir. (Tokyo).

[16] Pandey R, Caflisch L, Lodi A, Brenner AJ, Tiziani S (2017). Metabolomic signature of brain cancer. Molecular Carcinogenesis.

[17] Wolahan SM, Hirt D, Braas D, Glenn TC (2016). Role of Metabolomics in Traumatic Brain Injury Research. Neurosurgery Clinics of North America.

[18] Zheng F (2017). Plasma metabolomics profiles in rats with acute traumatic brain injury. PloS one.

[19] Botas A, Campbell HM, Han X, Maletic-Savatic M (2015). Metabolomics of neurodegenerative diseases. International Review of Neurobiology.

[20] Denihan, N. M. *et al*. Untargeted metabolomic analysis and pathway discovery in perinatal asphyxia and hypoxic-ischaemic encephalopathy. *Journal of Cerebral Blood Flow and Metabolism: Official Journal of the International Society of Cerebral Blood Flow and Metabolism*, 271678X17726502, 10.1177/0271678X17726502 (2017). 10.1177/0271678X17726502 PMC6311668 28840775

[21] Zhang Y (2018). Integrated Metabolomics and Proteomics Analysis of Hippocampus in a Rat Model of Depression. Neuroscience.

[22] Gonzalez-Riano C, Garcia A, Barbas C (2016). Metabolomics studies in brain tissue: A review. Journal of Pharmaceutical and Biomedical Analysis.

[23] Wang D, Kong J, Wu J, Wang X, Lai M (2017). GC-MS-based metabolomics identifies an amino acid signature of acute ischemic stroke. Neuroscience Letters.

[24] Liu P (2017). Discovery of Metabolite Biomarkers for Acute Ischemic Stroke Progression. Journal of Proteome Research.

[25] Liu M (2015). Potential of serum metabolites for diagnosing post-stroke cognitive impairment. Molecular Biosystems.

[26] Botosoa EP (2012). NMR metabolomic of frontal cortex extracts: First study comparing two neurodegenerative diseases, Alzheimer disease and amyotrophic lateral sclerosis. Irbm.

[27] Gonzalez-Dominguez R, Garcia-Barrera T, Vitorica J, Gomez-Ariza JL (2015). Metabolomic screening of regional brain alterations in the APP/PS1 transgenic model of Alzheimer’s disease by direct infusion mass spectrometry. Journal of Pharmaceutical and Biomedical Analysis.

[28] Musgrove RE (2014). The metabolomics of alpha-synuclein (SNCA) gene deletion and mutation in mouse brain. Metabolomics.

[29] Smith ML, Auer RN, Siesjo BK (1984). The density and distribution of ischemic brain injury in the rat following 2-10 min of forebrain ischemia. Acta Neuropathologica.

[30] Sato-Maeda M (2017). Transient Global Cerebral Ischemia Induces RNF213, a Moyamoya Disease Susceptibility Gene, in Vulnerable Neurons of the Rat Hippocampus CA1 Subregion and Ischemic. Cortex. Journal of Stroke and Cerebrovascular Diseases: the Official Journal of National Stroke Association.

[31] Miyawaki S (2016). Imaging mass spectrometry detects dynamic changes of phosphatidylcholine in rat hippocampal CA1 after transient global ischemia. Neuroscience.

[32] Asai A (2002). Selective proteasomal dysfunction in the hippocampal CA1 region after transient forebrain ischemia. Journal of Cerebral Blood Flow and Metabolism: Official Journal of the International Society of Cerebral Blood Flow and Metabolism.

[33] Sugawara T, Fujimura M, Morita-Fujimura Y, Kawase M, Chan PH (1999). Mitochondrial release of cytochrome c corresponds to the selective vulnerability of hippocampal CA1 neurons in rats after transient global cerebral ischemia. The Journal of Neuroscience: The Official Journal of the Society for Neuroscience.

[34] Gu L (2013). Early activation of nSMase2/ceramide pathway in astrocytes is involved in ischemia-associated neuronal damage via inflammation in rat hippocampi. J. Neuroinflammation.

[35] Hamazaki K, Kim HY (2013). Differential modification of the phospholipid profile by transient ischemia in rat hippocampal CA1 and CA3 regions. Prostaglandins Leukot Essent Fatty Acids.

[36] Chong J (2018). MetaboAnalyst 4.0: towards more transparent and integrative metabolomics analysis. Nucleic Acids Research.

[37] Atlasi MA, Naderian H, Noureddini M, Fakharian E, Azami A (2013). Morphology of Rat Hippocampal CA1 Neurons Following Modified Two and Four-Vessels Global Ischemia Models. Archives of Trauma Research.

[38] Abe K (1995). Ischemic delayed neuronal death. A mitochondrial hypothesis. Stroke.

[39] Andine P, Jacobson I, Hagberg H (1988). Calcium uptake evoked by electrical stimulation is enhanced postischemically and precedes delayed neuronal death in CA1 of rat hippocampus: involvement of N-methyl-D-aspartate receptors. Journal of Cerebral Blood Flow and Metabolism: Official Journal of the International Society of Cerebral Blood Flow and Metabolism.

[40] Suzuki R, Yamaguchi T, Li CL, Klatzo I (1983). The effects of 5-minute ischemia in Mongolian gerbils: II. Changes of spontaneous neuronal activity in cerebral cortex and CA1 sector of hippocampus. Acta Neuropathologica.

[41] Zhang T (2016). Metabolomic investigation of regional brain tissue dysfunctions induced by global cerebral ischemia. BMC Neurosci.

[42] Pang B (2012). Defects in purine nucleotide metabolism lead to substantial incorporation of xanthine and hypoxanthine into DNA and RNA. Proceedings of the National Academy of Sciences of the United States of America.

[43] Wang L (2016). Mitochondrial purine and pyrimidine metabolism and beyond. Nucleosides, Nucleotides & Nucleic Acids.

[44] Kawakita M, Ishida N, Miura N, Sun-Wada GH, Yoshioka S (1998). Nucleotide sugar transporters: elucidation of their molecular identity and its implication for future studies. J. Biochem.

[45] Nakajima K (2013). Mass isotopomer analysis of metabolically labeled nucleotide sugars and N- and O-glycans for tracing nucleotide sugar metabolisms. Mol. Cell Proteomics.

[46] Liu L, Hirschberg CB (2013). Developmental diseases caused by impaired nucleotide sugar transporters. Glycoconj J..

[47] Lee DK (2016). Metabolic response induced by parasitic plant-fungus interactions hinder amino sugar and nucleotide sugar metabolism in the host. Scientific Reports.

[48] Richter K, Haslbeck M, Buchner J (2010). The heat shock response: life on the verge of death. Molecular cell.

[49] Theodoropoulou S (2013). Aminoimidazole carboxamide ribonucleotide (AICAR) inhibits the growth of retinoblastoma *in vivo* by decreasing angiogenesis and inducing apoptosis. PloS one.

[50] Mukhopadhyay S, Chatterjee A, Kogan D, Patel D, Foster DA (2015). 5-Aminoimidazole-4-carboxamide-1-beta-4-ribofuranoside (AICAR) enhances the efficacy of rapamycin in human cancer cells. Cell Cycle.

[51] Webley SD, Welsh SJ, Jackman AL, Aherne GW (2001). The ability to accumulate deoxyuridine triphosphate and cellular response to thymidylate synthase (TS) inhibition. British Journal of Cancer.

[52] Sato K (2018). Metabolomic changes in the mouse retina after optic nerve injury. Scientific Reports.

