# Supplementary material for: Metabolic basis of neuronal vulnerability to ischemia; an in vivo untargeted metabolomics approach
Source: Sci Rep. 2020 Apr 16;10:6507. doi: 10.1038/s41598-020-63483-w (PMC7162929; doi:10.1038/s41598-020-63483-w)
Supplement: Supplementary file 1 — Supplementary data. [file 41598_2020_63483_MOESM1_ESM.pdf]

Metabolic basis of neuronal vulnerability to ischemia; an *in vivo* untargeted metabolomics approach

Sherif Rashad, MD, PhD[1,2]*. Daisuke Saigusa, PhD[3,4]. Takahiro Yamazaki[5], Yotaro Matsumoto[5], Yoshihisa Tomioka[5], Ritsumi Saito[3,4]. Akira Uruno, MD, PhD[3,4]. Kuniyasu Niizuma, MD, PhD[1,2,6]*. Masayuki Yamamoto, MD, PhD[3,4]. Teiji Tominaga, MD, PhD[2].

1- Department of Neurosurgical Engineering and Translational Neuroscience, Tohoku University Graduate School of Medicine, Sendai, Japan.
2- Department of Neurosurgery, Tohoku University Graduate School of Medicine, Sendai, Japan.
3- Department of Integrative Genomics, Tohoku Medical Megabank Organization, Sendai, Japan.
4- Medical Biochemistry, Tohoku University School of Medicine, Sendai, Japan.
5- Laboratory of Oncology, Pharmacy Practice and Sciences, Graduate School of Pharmaceutical Sciences, Tohoku University
6- Department of Neurosurgical Engineering and Translational Neuroscience, Graduate School of Biomedical Engineering, Tohoku University, Sendai, Japan.

***Corresponding author:**

Corresponding author 1: Sherif Rashad, MD, PhD.

e-mail: sherif@nsg.med.tohoku.ac.jp , sherif_rashad@hotmail.com

Corresponding author 2: Kuniyasu Niizuma, M.D., Ph.D.

E-mail: niizuma@nsg.med.tohoku.ac.jp

Supplemental information

Supplementary Figure 1: Principle component analysis (PCA) comparison between CA1 and CA3 1 hour after transient global cerebral ischemia (tGCI). Significant differences are evident between both sub-regions 1 hour after tGCI, much earlier than any cellular changes.

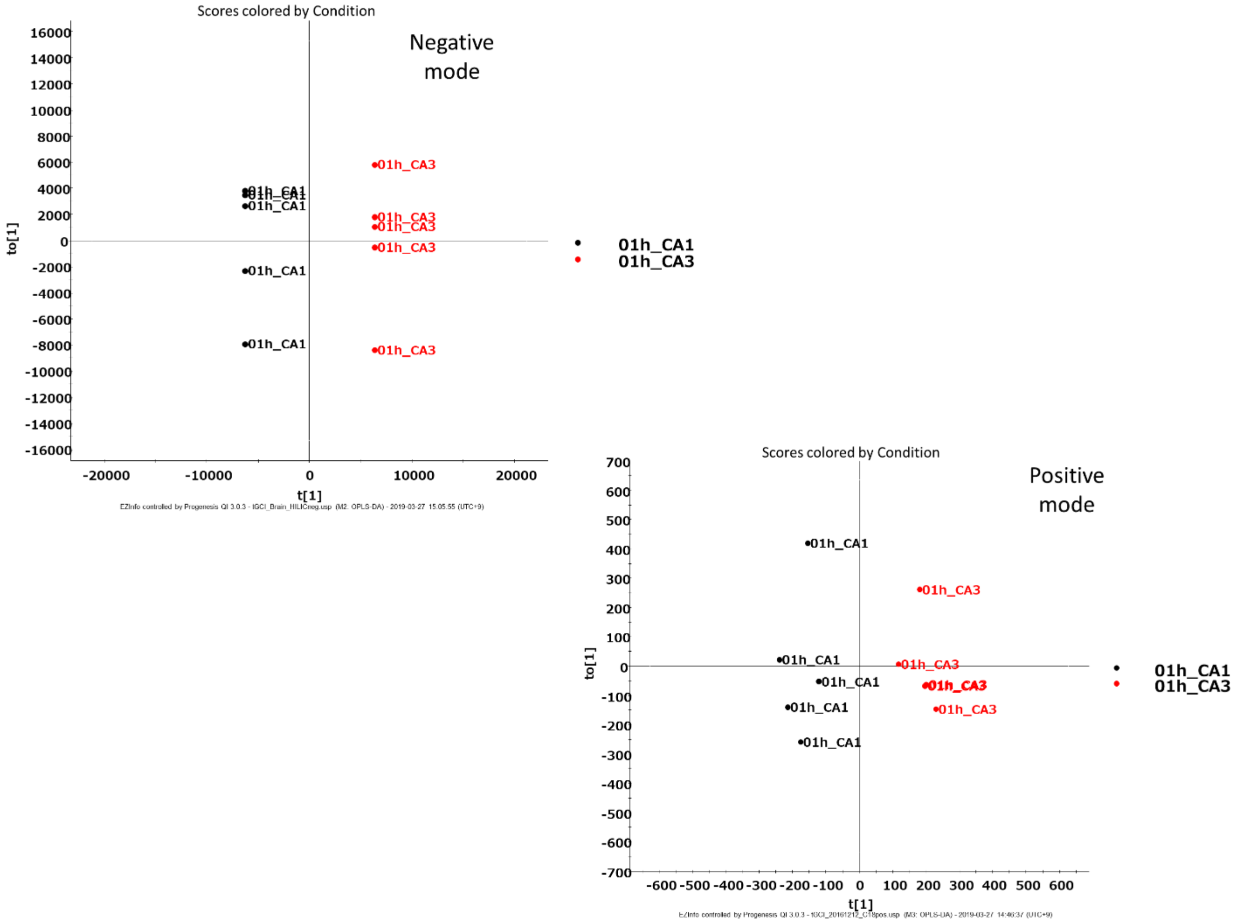

Supplementary Figure 2: Principle component analysis comparison between CA1 and CA3 6 hours after tGCI. Significant differences still exist between both sub-regions 6 hours after tGCI.

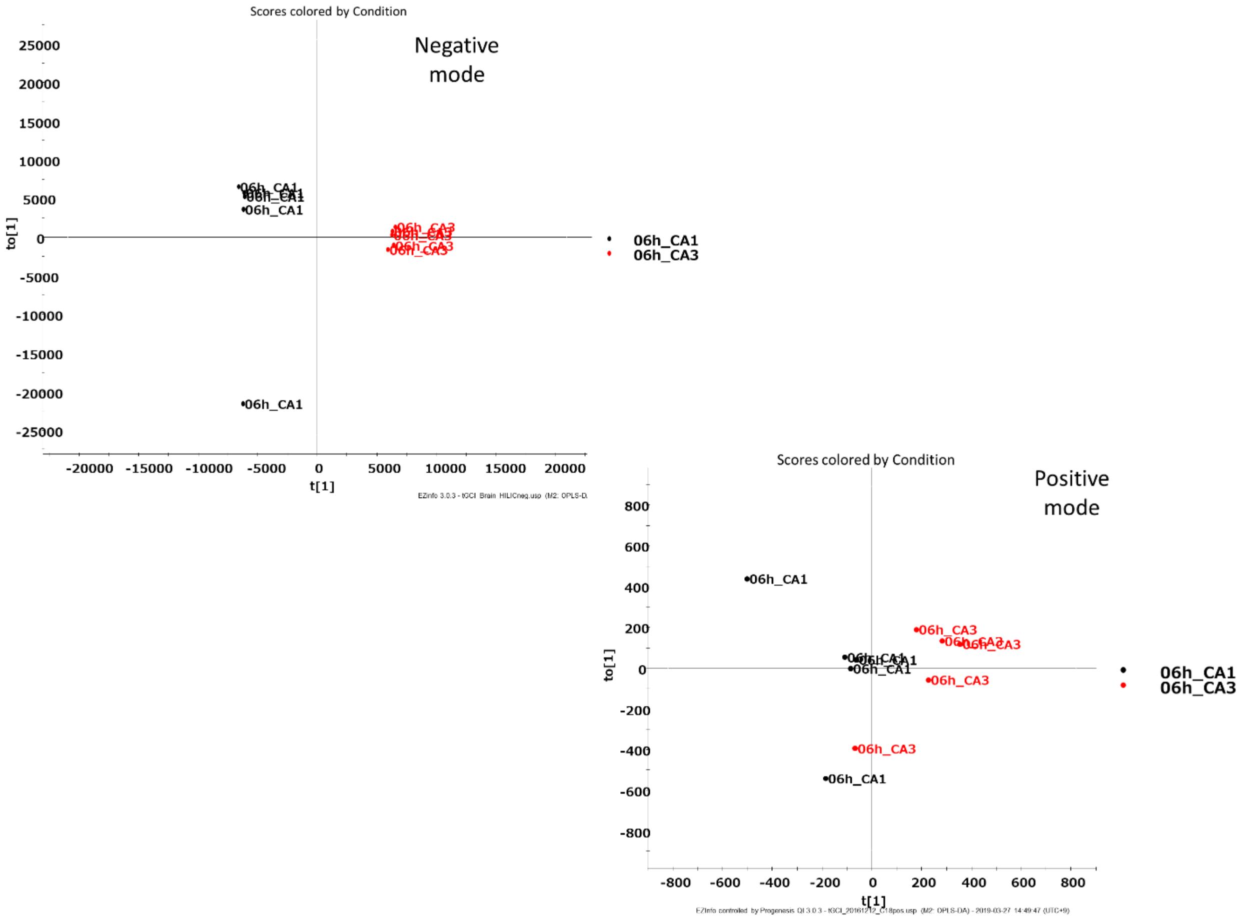

Supplementary Figure 3: Principle component analysis comparison between CA1 and CA3 24 hours after tGCI. 24 hours after tGCI, the pattern of metabolome regulation converges in both subregions, evident as less separation on PCA analysis.

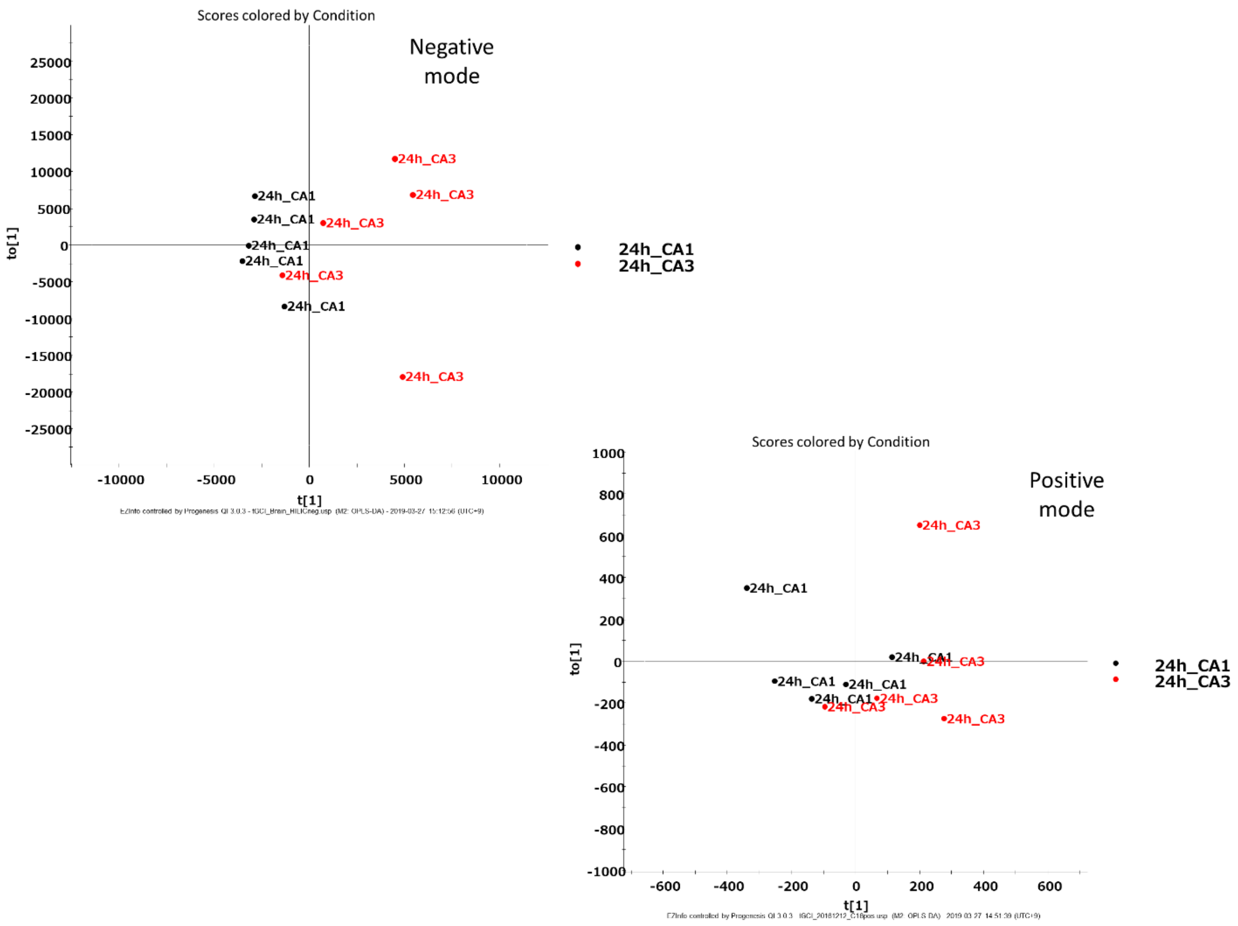

Supplementary Figure 4: Principle component analysis comparison between CA1 and CA3 48 hours after tGCI. As with the 24 hours mark, no significant differences exist between both regions at this time point.

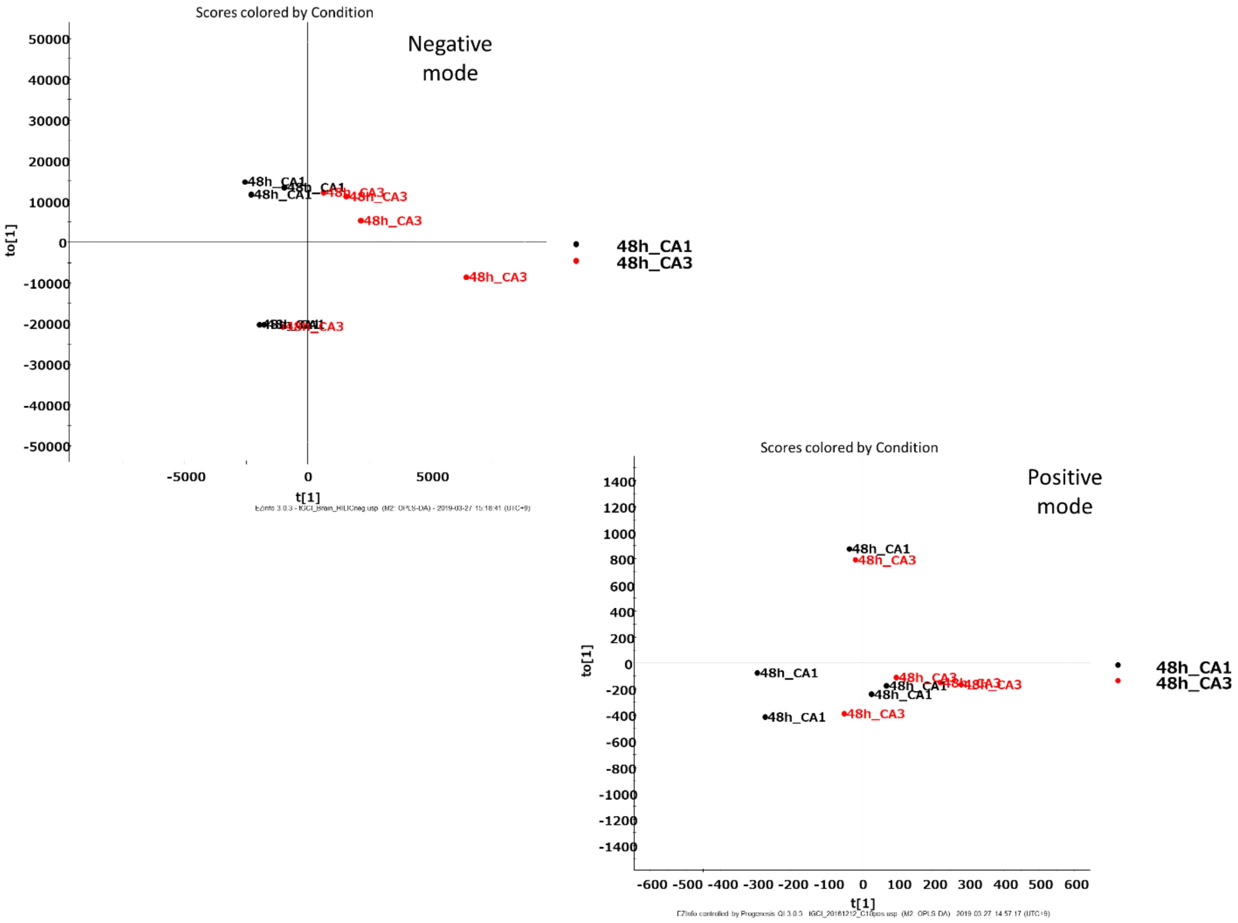

Supplementary Figure 5: Principle component analysis comparison between CA1 and CA3 72 hours after tGCI. At this time-point apoptosis is consistently observed in CA1. There appears to be some differences between both regions, but less sharp than at earlier time points.

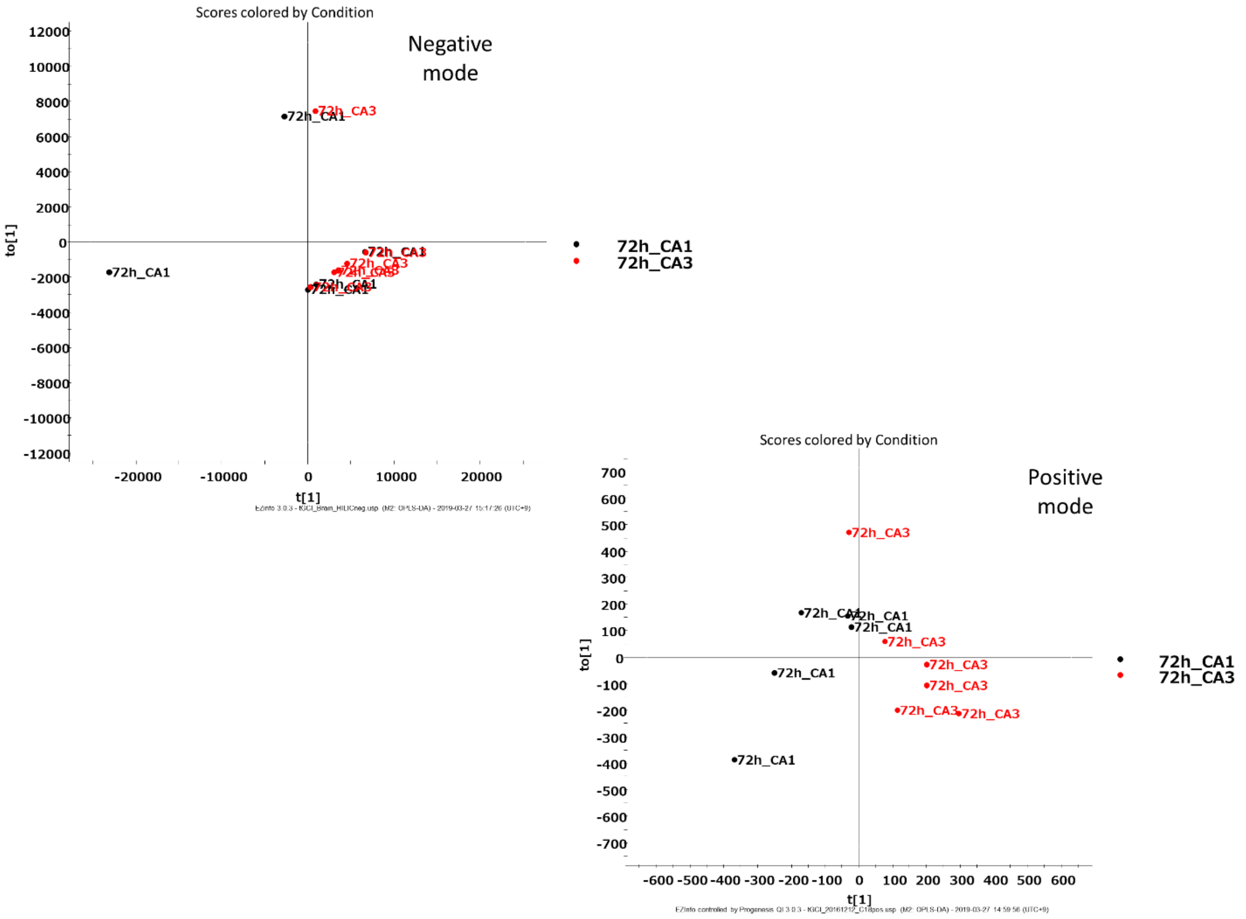

Supplementary Table 1: Significant and successfully annotated compounds in CA1 after ANOVA and Levene test.

**Compound name**	***tR*_m/z**	**Detected**	**Formula**	**Neutral**	**Adduct**	**Theoritical m/z**	**Delta ppm**	**Status**	**Mode**	**Pubchem ID**	**f.value**	**p.value**	**FDR**
Guanosine	4.17_282.0836m/z	282.0836	C10H13N5O5	283.0917	M-H	282.0844	2.8	C	Negative	6802	21.278	4.15E-08	3.65E-05
N-omega-(ADP-D-ribosyl)-L-arginine (ADP-Ribosyl-L-arginine)	3.98_698.1797m/z	698.1797	C21H35N9O15P2	715.1728	M+H-H2O	698.1701	-13.8	P	Positive	46173712	15.367	8.26E-07	9.93E-05
gamma-Glutamylglutamic acid	5.36_275.0879m/z	275.0879	C10H16N2O7	276.0958	M-H	275.0885	2.2	P	Negative	92865	14.727	1.20E-06	0.00016
Xanthine	3.50_151.0257m/z	151.0257	C5H4N4O2	152.0334	M-H	151.0261	2.6	P	Negative	1188	14.592	1.30E-06	0.00016
5-Aminoimidazole-4-carboxamide	4.12_109.0510m/z	109.0510	C4H6N4O	126.0542	M+H-H2O	109.0515	4.6	P	Positive	9679	14.495	1.38E-06	0.00015
Adenine	3.19_134.0468m/z	134.0468	C5H5N5	135.0545	M-H	134.0472	3.0	C	Negative	190	13.797	2.11E-06	0.00017
Adenosine	3.20_266.0888m/z	266.0888	C10H13N5O4	267.0968	M-H	266.0895	2.6	C	Negative	60961	13.671	2.28E-06	0.00017
Uracil	2.53_111.0196m/z	111.0196	C4H4N2O2	112.0273	M-H	111.0200	3.6	C	Negative	1174	13.383	2.73E-06	0.00017
Cysteine	4.81_120.0121m/z	120.0121	C3H7NO2S	121.0197	M-H	120.0125	3.3	C	Negative	5862	13.309	2.86E-06	0.00017
Stachyose	3.99_705.1913m/z	705.1913	C24H42O21	666.2219	M+K	705.1850	-8.9	P	Positive	439531	13.09	3.29E-06	0.00032
Acetyl-L-methionine	3.12_190.0539m/z	190.0539	C7H13NO3S	191.0616	M-H	190.0543	2.1	P	Negative	448580	13.081	3.31E-06	0.00017
S-formylglutathione	3.20_334.0762m/z	334.0762	C11H17N3O7S	335.0787	M-H	334.0714	-14.4	P	Negative	189122	12.427	5.08E-06	0.00022
gamma-Glutamylglutamine	5.08_274.1039m/z	274.1039	C10H17N3O6	275.1117	M-H	274.1045	2.2	P	Negative	150914	11.61	8.88E-06	0.00029
Inosine	3.74_267.0728m/z	267.0728	C10H12N4O5	268.0808	M-H	267.0735	2.6	C	Negative	6021	10.821	1.56E-05	0.00043
hypoxanthine	3.34_135.0309m/z	135.0309	C5H4N4O	136.0385	M-H	135.0312	2.2	C	Negative	790	10.735	1.66E-05	0.00044
D-Ribose 5-phosphate	5.16_229.0112m/z	229.0112	C5H11O8P	230.0192	M-H	229.0119	3.1	P	Negative	439236	10.631	1.79E-05	0.00045
Cytosine	3.97_94.0399m/z	94.0399	C4H5N3O	111.0433	M+H-H2O	94.0406	7.4	C	Positive	597	10.076	2.72E-05	0.00151
Glutathione	3.19_288.0707m/z	288.0707	C10H17N3O6S	307.0838	M-H20-H	288.0654	-18.4	C	Negative	124886	9.7783	3.42E-05	0.00073

Uridine diphosphate-N-acetylgalactosamine	5.43_646.0499m/z	646.0499	C17H27N3O17P2	607.0816	M+K	646.0447	-8.0	P	Positive	445675	9.2986	5.00E-05	0.00193
4-phosphopantothenoylcysteine	3.33_401.0817m/z	401.0817	C12H23N2O9PS	402.0862	M-H	401.0789	-7.0	P	Negative	440304	9.0327	6.20E-05	0.00112
L-histidine	4.89_154.0617m/z	154.0617	C6H9N3O2	155.0695	M-H	154.0622	3.2	C	Negative	6274	9.0253	6.23E-05	0.00112
4-Aminobutanal	0.98_70.0650m/z	70.0650	C4H9NO	87.0684	M+H-H2O	70.0657	10.0	P	Positive	118	8.7295	7.96E-05	0.00264
UDP-N-Acetyl-D-hexosamine	3.97_590.0798m/z	590.0798	C17H27N3O17P2	607.0816	M+H-H2O	590.0789	-1.5	P	Positive	445675	8.2875	0.0001156	0.00321
Glutamylglutamic acid	1.44_277.1032m/z	277.1032	C10H16N2O7	276.0958	M+H	277.1030	-0.7	P	Positive	439500	8.2838	0.000116	0.00321
Cytidine	4.01_242.0777m/z	242.0777	C9H13N3O5	243.0855	M-H	242.0782	2.1	C	Negative	6175	8.0655	0.0001401	0.00209
Cytidine-5'-monophosphate-5-N-acetylneuraminic acid	3.99_597.1428m/z	597.1428	C20H31N4O16P	614.1473	M+H-H2O	597.1446	3.0	P	Positive	448209	7.6742	0.000198	0.00443
Nicotinamide adenine dinucleotide (NAD+)	2.95_664.1144m/z	664.1144	C21H28N7O14P2	664.1169	M+H	664.1169	3.8	C	Positive	5892	7.5154	0.0002285	0.00499
Aconitic acid (cis)	5.48_173.0086m/z	173.0086	C6H6O6	174.0164	M-H	173.0092	3.5	C	Negative	643757	7.4715	0.0002377	0.00297
Arginine	7.23_173.1039m/z	173.1039	C6H14N4O2	174.1117	M-H	173.1044	2.9	C	Negative	232	7.4325	0.0002463	0.00297
Gluconic acid	3.75_195.0505m/z	195.0505	C6H12O7	196.0583	M-H	195.0510	2.6	C	Negative	10690	7.2899	0.0002807	0.00322
Uracil	3.31_111.0197m/z	111.0197	C4H4N2O2	112.0273	M-H	111.0200	2.7	C	Negative	1174	6.7372	0.000472	0.00477
Dopamine 4-O-sulfate	3.50_214.0213m/z	214.0213	C8H11NO5S	233.0358	M-H20-H	214.0174	-18.2	P	Negative	123932	6.7158	0.0004818	0.00481
6-Hydroxyhexanoic acid	2.62_131.0709m/z	131.0709	C6H12O3	132.0786	M-H	131.0714	3.8	P	Negative	14490	6.4548	0.0006206	0.00587
5-hydroxypentanoic acid	2.83_117.0554m/z	117.0554	C5H10O3	118.0630	M-H	117.0557	2.6	P	Negative	25945	6.3331	0.0006996	0.00634
Uridine	3.31_243.0616m/z	243.0616	C9H12N2O6	244.0695	M-H	243.0623	2.9	C	Negative	6029	6.2819	0.000736	0.0064
Phosphohydroxypyruvic Acid	5.26_164.9565m/z	164.9565	C3H5O7P	183.9773	M-H20-H	164.9589	14.5	P	Negative	105	5.6741	0.0013654	0.00949
N-acetyl-L-phenylalanine	2.77_206.0817m/z	206.0817	C11H13NO3	207.0895	M-H	206.0823	2.9	P	Negative	74839	5.6696	0.0013718	0.00949
D-mannitol	4.56_181.0713m/z	181.0713	C6H14O6	182.0790	M-H	181.0718	2.8	P	Negative	6251	5.6368	0.0014195	0.00967

Acetyl Glutamic Acid	3.06_188.0559m/z	188.0559	C7H11NO5	189.0637	M-H	188.0564	2.7	P	Negative	70914	5.5883	0.0014933	0.01002
Methylcitric acid	5.55_205.0348m/z	205.0348	C7H10O7	206.0427	M-H	205.0354	2.9	P	Negative	515	5.5705	0.0015215	0.01006
N-Formyl-L-glutamic acid	3.06_156.0298m/z	156.0298	C6H9NO5	175.0481	M-H20-H	156.0297	-0.6	P	Negative	439376	5.4721	0.0016878	0.01067
L-tryptophan	4.06_203.0821m/z	203.0821	C11H12N2O2	204.0899	M-H	203.0826	2.5	C	Negative	6305	5.4405	0.0017451	0.01088
N-Acetyl-S-(N-methylcarbamoyl)cysteine	4.56_219.0449m/z	219.0449	C7H12N2O4S	220.0518	M-H	219.0445	-1.8	P	Negative	108218	5.402	0.0018181	0.01118
1-(beta-D-Ribofuranosyl)-1,4-dihydronicotinamide (reduced nicotinamide riboside)	1.26_295.0662m/z	295.0662	C11H16N2O5	256.1059	M+K	295.0691	9.8	P	Positive	11507134	5.2273	0.0021922	0.02295
Guanosine Diphosphate Mannose	3.97_588.0831m/z	588.0831	C16H25N5O16P2	605.0772	M+H-H2O	588.0745	-14.6	P	Positive	18396	5.2152	0.0022209	0.02295
Lipoyl-amp	4.98_534.0881m/z	534.0881	C18H26N5O8PS2	535.0960	M-H	534.0888	1.3	P	Negative	23724672	5.2144	0.0022229	0.01311
Cytidine-5'-diphosphate	3.97_386.0156m/z	386.0156	C9H15N3O11P2	403.0182	M+H-H2O	386.0155	-0.3	P	Positive	6132	5.1558	0.0023684	0.02421
N-acetyl-L-leucine	2.81_172.0975m/z	172.0975	C8H15NO3	173.1052	M-H	172.0979	2.3	P	Negative	70912	5.1503	0.0023827	0.01363
P1,P4-Bis(5'-xanthosyl) tetraphosphate	6.12_892.9875m/z	892.9875	C20H26N8O23P4	870.0061	M+Na	892.9953	8.7	P	Positive	440322	5.023	0.0027378	0.02631
N-acetylneuraminic acid	4.65_290.0874m/z	290.0874	C11H19NO9	309.1060	M-H20-H	290.0876	0.7	P	Negative	445063	4.9249	0.0030499	0.01596
Biotinyl-5-AMP	3.97_556.1410m/z	556.1410	C20H28N7O9PS	573.1407	M+H-H2O	556.1380	-5.4	P	Positive	440839	4.8455	0.0033308	0.02992
Nicotinic acid	1.41_121.0403m/z	121.0403	C6H8N2O2	140.0586	M-H20-H	121.0402	-0.8	P	Negative	938	4.6509	0.004143	0.01947
isocitric acid or citric acid	5.64_191.0193m/z	191.0193	C6H8O7	192.0270	M-H	191.0197	2.1	C	Negative	1198 or 311	4.6127	0.0043259	0.0197
L-phenylalanine	3.84_164.0713m/z	164.0713	C9H11NO2	165.0790	M-H	164.0717	2.4	C	Negative	6140	4.4196	0.0053946	0.02269
Cytidine Diphosphate Ethanolamine	5.26_445.0521m/z	445.0521	C11H20N4O11P2	446.0604	M-H	445.0531	2.2	P	Negative	123727	4.2265	0.0067498	0.02668
Aspartic Acid	4.98_114.0193m/z	114.0193	C4H7NO4	133.0375	M-H20-H	114.0191	-1.8	C	Negative	5960	4.2204	0.0067985	0.02668

N-acetyl-l-aspartic aid	3.40_156.0298m/z	156.0298	C6H9NO5	175.0481	M-H20-H	156.0297	-0.6	P	Negative	65065	4.1683	0.0072267	0.02774
Glycerol 3-phosphate	5.04_171.0060m/z	171.0060	C3H9O6P	172.0137	M-H	171.0064	2.3	P	Negative	754	4.1563	0.0073297	0.02789
N-acetyl-l-glutamic acid	3.40_188.0559m/z	188.0559	C7H11NO5	189.0637	M-H	188.0564	2.7	P	Negative	70914	4.0676	0.0081391	0.03057
Leucine	3.90_130.0869m/z	130.0869	C6H13NO2	131.0946	M-H	130.0874	3.8	C	Negative	6106	3.8306	0.010807	0.03942
5-amino-6-(5-phospho-D-ribitylamino)uracil	5.15_355.0669m/z	355.0669	C9H17N4O9P	356.0733	M-H	355.0660	-2.5	P	Negative	18666812	3.7137	0.012456	0.04469
N-Acetylornithine or Theanine	4.65_173.0927m/z	173.0927	C7H14N2O3	174.1004	M-H	173.0932	2.9	P	Negative	439232 or 439378	3.6827	0.012936	0.04538
phosphoethanolamine	5.17_140.0114m/z	140.0114	C2H8NO4P	141.0191	M-H	140.0118	2.9	P	Negative	1015	3.6349	0.013716	0.04728
Phosphodimethylethanolamine	5.04_168.0427m/z	168.0427	C4H12NO4P	169.0504	M-H	168.0431	2.4	P	Negative	151438	3.5842	0.014599	0.04974

Abbreviations: C: confirmed by chemical standard. P: Putative annotation

Supplementary Table 2: Significant and successfully annotated compounds in CA3 after ANOVA.

**Compound name**	***tR*_m/z**	**Detected**	**Formula**	**Neutral**	**Adduct**	**Theoritical m/z**	**Delta ppm**	**Status**	**Mode**	**PubChem ID**	**f.value**	**p.value**	**FDR**
gamma-Glutamylglutamic acid	5.36_275.0879 m/z	275.0879	C10H16N2O7	276.0958	M-H	275.0885	2.2	P	Negative	92865	24.07	8.22E-09	2.42E-06
Glutathione	3.19_288.0707 m/z	288.0707	C10H17N3O6S	307.0838	M-H20-H	288.0654	-18.4	C	Negative	124886	20.433	4.19E-08	5.89E-06
Hypoxanthine	3.34_135.0309 m/z	135.0309	C5H4N4O	136.0385	M-H	135.0312	2.2	C	Negative	790	19.367	7.05E-08	6.94E-06
S-formylglutathione	3.20_334.0762 m/z	334.0762	C11H17N3O7S	335.0787	M-H	334.0714	-14.4	P	Negative	189122	18.007	1.42E-07	9.62E-06
Adenosine	3.20_266.0888 m/z	266.0888	C10H13N5O4	267.0968	M-H	266.0895	2.6	C	Negative	60961	15.875	4.59E-07	1.93E-05
Cytidine	4.01_242.0777 m/z	242.0777	C9H13N3O5	243.0855	M-H	242.0782	2.1	C	Negative	6175	15.185	6.89E-07	2.34E-05
gamma-Glutamylglutamine	5.08_274.1039 m/z	274.1039	C10H17N3O6	275.1117	M-H	274.1045	2.2	P	Negative	150914	15.12	7.16E-07	2.34E-05
N-acetylhexosamine 6-phosphate	5.06_300.0483 m/z	300.0483	C8H16NO9P	301.0563	M-H	300.0490	2.3	P	Negative	439219	13.855	1.57E-06	4.32E-05
N-Carbamoylsarcosine	5.01_131.0458 m/z	131.0458	C4H8N2O3	132.0535	M-H	131.0462	3.1	P	Negative	439375	12.875	2.97E-06	7.28E-05
Arginine	7.23_173.1039 m/z	173.1039	C6H14N4O2	174.1117	M-H	173.1044	2.9	C	Negative	232	12.692	3.36E-06	7.41E-05
D-hexose-1-phosphate	5.16_229.0112 m/z	229.0112	C5H11O8P	230.0192	M-H	229.0119	3.1	P	Negative	439236	12.536	3.73E-06	7.92E-05
N-Acetylornithine or Theanine	4.65_173.0927 m/z	173.0927	C7H14N2O3	174.1004	M-H	173.0932	2.9	P	Negative	439232 or 439378	11.352	8.60E-06	0.00014
L-(-)-Threonine	4.82_118.0506 m/z	118.0506	C4H9NO3	119.0582	M-H	118.0510	3.4	C	Negative	6288	10.871	1.23E-05	0.00017
N-Formyl-L-glutamic acid	3.06_156.0298 m/z	156.0298	C6H9NO5	175.0481	M-H20-H	156.0297	-0.6	P	Negative	439376	10.667	1.43E-05	0.00019
Acetyl Glutamic Acid	3.06_188.0559 m/z	188.0559	C7H11NO5	189.0637	M-H	188.0564	2.7	P	Negative	70914	10.554	1.56E-05	0.0002
Glutamylglutamic acid	1.44_277.1032 m/z	277.1032	C10H16N2O7	276.0958	M+H	277.1030	-0.7	P	Positive	439500	10.523	1.60E-05	0.00191

Cystathionine	5.44_221.0596 m/z	221.0596	C7H14N2O4S	222.0674	M-H	221.0602	2.7	C	Negative	834	10.454	1.68E-05	0.0002
Citrulline	5.10_174.0877 m/z	174.0877	C6H13N3O3	175.0957	M-H	174.0884	4.0	C	Negative	9750	10.437	1.70E-05	0.0002
Aspartic Acid	4.98_114.0193 m/z	114.0193	C4H7NO4	133.0375	M-H20-H	114.0191	-1.8	C	Negative	5960	10.423	1.72E-05	0.0002
Serine	5.06_104.0354 m/z	104.0354	C3H7NO3	105.0426	M-H	104.0353	-1.0	C	Negative	5951	10.24	1.98E-05	0.00021
5-hydroxypentanoic acid	2.83_117.0554 m/z	117.0554	C5H10O3	118.0630	M-H	117.0557	2.6	P	Negative	25945	10.045	2.31E-05	0.00022
Inosine	3.74_267.0728 m/z	267.0728	C10H12N4O5	268.0808	M-H	267.0735	2.6	C	Negative	6021	10.042	2.32E-05	0.00022
Xanthine	3.50_151.0257 m/z	151.0257	C5H4N4O2	152.0334	M-H	151.0261	2.6	P	Negative	1188	9.9078	2.58E-05	0.00024
Pantothenol	2.76_186.1131 m/z	186.1131	C9H19NO4	205.1314	M-H20-H	186.1130	-0.5	P	Negative	4678	9.1568	4.74E-05	0.00034
Uracil	3.31_111.0197 m/z	111.0197	C4H4N2O2	112.0273	M-H	111.0200	2.7	C	Negative	1174	9.0155	5.34E-05	0.00037
Fumaric acid or Maleic acid	5.17_115.0033 m/z	115.0033	C4H4O4	116.0110	M-H	115.0037	3.5	P	Negative	444972 or 444266	8.9203	5.78E-05	0.0004
L-Pyroglutamic acid	4.36_128.0349 m/z	128.0349	C5H7NO3	129.0426	M-H	128.0353	3.1	P	Negative	7405	8.861	6.08E-05	0.0004
L-Glutamic acid 5-phosphate	5.17_207.9987 m/z	207.9987	C5H10NO7P	227.0195	M-H20-H	208.0011	11.5	P	Negative	440099	8.5894	7.67E-05	0.00046
N-acetyl-l-glutamic acid	3.40_188.0559 m/z	188.0559	C7H11NO5	189.0637	M-H	188.0564	2.7	P	Negative	70914	8.5212	8.13E-05	0.00048
Homovanillic acid or Phenylpyruvic acid	2.62_163.0396 m/z	163.0396	C9H10O4	182.0579	M-H20-H	163.0395	-0.6	P	Negative	1738 or 997	8.4668	8.53E-05	0.00048
Cysteine	4.81_120.0121 m/z	120.0121	C3H7NO2S	121.0197	M-H	120.0125	3.3	C	Negative	5862	8.4538	8.62E-05	0.00048
Betalamic acid	3.57_192.0331 m/z	192.0331	C9H9NO5	211.0481	M-H20-H	192.0297	-17.7	P	Negative	5281176	8.2919	9.94E-05	0.00052
Phosphodimethylethanolamine	5.04_168.0427 m/z	168.0427	C4H12NO4P	169.0504	M-H	168.0431	2.4	P	Negative	151438	8.2907	9.95E-05	0.00052
N-acetyl-l-aspartic aid	3.40_156.0298 m/z	156.0298	C6H9NO5	175.0481	M-H20-H	156.0297	-0.6	P	Negative	65065	8.208	0.000107	0.00054
Homocarnosine	5.21_239.1143 m/z	239.1143	C10H16N4O3	240.1222	M-H	239.1150	2.9	P	Negative	10243361	8.202	0.000108	0.00054

Alanine	4.75_88.0401 m/z	88.0401	C3H7NO2	89.0477	M-H	88.0404	3.4	C	Negative	5950	8.0873	0.000119	0.00057
Guanosine	4.17_282.0836 m/z	282.0836	C10H13N5O5	283.0917	M-H	282.0844	2.8	C	Negative	6802	8.0805	0.00012	0.00057
D-Galactose	5.25_179.0556 m/z	179.0556	C6H12O6	180.0634	M-H	179.0561	2.8	P	Negative	6036	7.9003	0.000141	0.00063
2,5-Dichloro-4-oxohex-2-enedioate	7.78_206.9284 m/z	206.9284	C6H4Cl2O5	225.9436	M-H20-H	206.9252	-15.5	P	Negative	5282172	7.8883	0.000142	0.00063
phosphoethanolamine	5.17_140.0114 m/z	140.0114	C2H8NO4P	141.0191	M-H	140.0118	2.9	P	Negative	1015	7.881	0.000143	0.00063
Taurine	4.85_124.0070 m/z	124.0070	C2H7NO3S	125.0147	M-H	124.0074	3.2	C	Negative	1123	7.8734	0.000144	0.00063
Malic acid	5.20_133.0138 m/z	133.0138	C4H6O5	134.0215	M-H	133.0142	3.0	C	Negative	525	7.7747	0.000158	0.00067
2-methylpropanedioic acid	7.71_116.9592 m/z	116.9592	C4H8Se	135.9791	M-H20-H	116.9607	12.8	P	Negative	487	7.7372	0.000163	0.00068
Dopachrome	3.27_174.0225 m/z	174.0225	C9H7NO4	193.0375	M-H20-H	174.0191	-19.5	P	Negative	5459802	7.6416	0.000178	0.00072
N-acetylneuraminic acid	4.80_308.0980 m/z	308.0980	C11H19NO9	309.1060	M-H	308.0987	2.3	P	Negative	439197	7.5554	0.000193	0.00076
PE(40:6)	2.48_790.5376 m/z	790.5376	C45H78NO8P	791.5465	M-H	790.5392	2.0	P	Negative	9546798	7.3997	0.000223	0.00081
Ascorbic acid or Glucuronic acid	4.73_175.0243 m/z	175.0243	C6H10O7	194.0427	M-H20-H	175.0243	0.0	P	Negative	54670067 or 94715	7.2985	0.000246	0.00087
Neuraminic acid	4.37_266.0874 m/z	266.0874	C9H17NO8	267.0954	M-H	266.0881	2.6	P	Negative	441037	7.2355	0.000261	0.00091
N-acetylneuraminic acid	4.65_290.0874 m/z	290.0874	C11H19NO9	309.1060	M-H20-H	290.0876	0.7	P	Negative	445063	7.1517	0.000282	0.00094
N-acetyl-D-glucosamine 6 phosphate	4.82_300.0387 m/z	300.0387	C8H15NO9S	301.0468	M-H	300.0395	2.7	P	Negative	440996	6.9086	0.000357	0.00111
N-acetyl-l-glutamic acid	4.89_188.0559 m/z	188.0559	C7H11NO5	189.0637	M-H	188.0564	2.7	P	Negative	70914	6.7077	0.000434	0.00129
Phenylacetaldehyde	4.21_103.0546 m/z	103.0546	C8H8O	120.0575	M+H-H2O	103.0548	1.9	P	Positive	998	6.4278	0.000573	0.01328
L-Xylonic acid	4.73_165.0399 m/z	165.0399	C5H10O6	166.0477	M-H	165.0405	3.6	P	Negative	6971043	6.2114	0.000714	0.00184
Lipoyl-amp	4.98_534.0881 m/z	534.0881	C18H26N5O8PS2	535.0960	M-H	534.0888	1.3	P	Negative	23724672	6.2065	0.000717	0.00184

Xanthylic acid	5.25_345.0196 m/z	345.0196	C10H13N4O9P	364.0420	M-H20-H	345.0236	11.6	P	Negative	73323	6.0619	0.000832	0.00203
4-Phosphopantothenoylcysteine	3.33_401.0817 m/z	401.0817	C12H23N2O9PS	402.0862	M-H	401.0789	-7.0	P	Negative	440304	6.0538	0.000839	0.00204
Aspartic acid	5.01_132.0298 m/z	132.0298	C4H7NO4	133.0375	M-H	132.0302	3.0	C	Negative	5960	6.0303	0.00086	0.00206
L-threonic acid	4.52_135.0295 m/z	135.0295	C4H8O5	136.0372	M-H	135.0299	3.0	C	Negative	5460407	5.9882	0.000898	0.00212
N-Acetyl-L-phenylalanine	2.77_206.0817 m/z	206.0817	C11H13NO3	207.0895	M-H	206.0823	2.9	P	Negative	74839	5.9627	0.000922	0.00215
UDP-GlcNAc	5.43_646.0499 m/z	646.0499	C17H27N3O17P2	607.0816	M+K	646.0447	-8.0	P	Positive	445675	5.9625	0.000922	0.01875
Creatinine	3.29_112.0513 m/z	112.0513	C4H9N3O2	131.0695	M-H20-H	112.0511	-1.8	C	Negative	588	5.8696	0.001016	0.00233
2-hydroxyglutaric acid	5.07_147.0295 m/z	147.0295	C5H8O5	148.0372	M-H	147.0299	2.7	P	Negative	43	5.7844	0.001112	0.0025
Hexose 1-phosphate	5.36_259.0217 m/z	259.0217	C6H13O9P	260.0297	M-H	259.0224	2.7	P	Negative	123912	5.7548	0.001147	0.00257
Cytidine-5'-monophosphate-5-N-acetylneuraminic acid	3.99_597.1428 m/z	597.1428	C20H31N4O16P	614.1473	M+H-H2O	597.1446	3.0	P	Positive	448209	5.633	0.001305	0.02322
Glycerol 3-phosphate	5.04_171.0060 m/z	171.0060	C3H9O6P	172.0137	M-H	171.0064	2.3	P	Negative	754	5.6209	0.001322	0.00284
D-4-Hydroxy-2-oxoglutarate	3.73_142.9981 m/z	142.9981	C5H6O6	162.0164	M-H20-H	142.9980	-0.7	P	Negative	440853	5.4529	0.001584	0.00323
Glutathione	4.91_306.0758 m/z	306.0758	C10H17N3O6S	307.0838	M-H	306.0765	2.3	C	Negative	124886	5.4233	0.001636	0.00332
PE(38:6)	2.51_762.5060 m/z	762.5060	C43H74NO8P	763.5152	M-H	762.5079	2.5	P	Negative	9546799	5.362	0.001748	0.00352
succinic acid	3.73_99.0090 m/z	99.0090	C4H6O4	118.0266	M-H20-H	99.0082	-8.1	C	Negative	1110	5.3139	0.001843	0.00367
N-Acetyl-D-hexosamine-6-phosphate	5.12_282.0353 m/z	282.0353	C8H16NO9P	301.0563	M-H20-H	282.0379	9.2	P	Negative	440996	5.1766	0.002143	0.00419
5-Aminoimidazole ribonucleotide	3.86_276.0353 m/z	276.0353	C8H14N3O7P	295.0569	M-H20-H	276.0385	11.6	P	Negative	161500	5.0724	0.002406	0.00454
Glycerophosphatidylethanolamine	5.12_214.0481 m/z	214.0481	C5H14NO6P	215.0559	M-H	214.0486	2.3	P	Negative	123874	4.8573	0.003064	0.00543

Dopamine 4-O-sulfate	3.50_214.0213 m/z	214.0213	C8H11NO5S	233.0358	M-H20-H	214.0174	-18.2	P	Negative	123932	4.7323	0.003533	0.00606
D-Ribose 5-phosphate	4.66_211.0009 m/z	211.0009	C5H11O8P	230.0192	M-H20-H	211.0008	-0.5	P	Negative	439236	4.0215	0.008162	0.01207

Abbreviations: C: confirmed by chemical standard. P: Putative annotation

Supplementary table 3: Pathway analysis in CA1 using MetaboAnalyst

**Pathway**	**Total**	**Expected**	**Hits**	**Raw p**	**-logP**	**Holm adjust**	**FDR**	**Impact**
Purine metabolism	66	2.0994	9	0.000146	8.835	0.012227	0.012227	0.05958
Pantothenate and CoA biosynthesis	19	0.60437	4	0.00246	6.0074	0.20422	0.093769	0.17857
Aminoacyl-tRNA biosynthesis	48	1.5268	6	0.003349	5.6991	0.27461	0.093769	0
beta-Alanine metabolism	21	0.66799	3	0.026865	3.6169	1	0.5499	0
Pyrimidine metabolism	39	1.2406	4	0.032732	3.4194	1	0.5499	0.1142
Arginine biosynthesis	14	0.44533	2	0.070701	2.6493	1	0.94058	0
Nicotinate and nicotinamide metabolism	15	0.47714	2	0.079962	2.5262	1	0.94058	0
Histidine metabolism	16	0.50895	2	0.089579	2.4126	1	0.94058	0.22131
Amino sugar and nucleotide sugar metabolism	37	1.1769	3	0.11039	2.2038	1	0.99381	0.07042
Phenylalanine, tyrosine and tryptophan biosynthesis	4	0.12724	1	0.12141	2.1086	1	0.99381	0.5
Citrate cycle (TCA cycle)	20	0.63618	2	0.13101	2.0325	1	0.99381	0.09501
Pentose phosphate pathway	21	0.66799	2	0.14197	1.9521	1	0.99381	0.05183
Thiamine metabolism	7	0.22266	1	0.20287	1.5952	1	1	0
Glutathione metabolism	28	0.89066	2	0.22291	1.501	1	1	0.25939
Alanine, aspartate and glutamate metabolism	28	0.89066	2	0.22291	1.501	1	1	0.3101
Valine, leucine and isoleucine biosynthesis	8	0.25447	1	0.22835	1.4769	1	1	0

Taurine and hypotaurine metabolism	8	0.25447	1	0.22835	1.4769	1	1	0
Glyoxylate and dicarboxylate metabolism	32	1.0179	2	0.27083	1.3063	1	1	0.02381
Biotin metabolism	10	0.31809	1	0.27693	1.284	1	1	0.15
Cysteine and methionine metabolism	33	1.0497	2	0.28285	1.2629	1	1	0.09687
Glycine, serine and threonine metabolism	34	1.0815	2	0.29485	1.2213	1	1	0.02247
Glycerophospholipid metabolism	36	1.1451	2	0.31879	1.1432	1	1	0.0514
Phenylalanine metabolism	12	0.38171	1	0.32251	1.1316	1	1	0.35714
Fructose and mannose metabolism	18	0.57256	1	0.44302	0.81414	1	1	0.10766
Sphingolipid metabolism	21	0.66799	1	0.49513	0.70293	1	1	0.0142
Galactose metabolism	27	0.85885	1	0.58544	0.53539	1	1	0.05832
Arginine and proline metabolism	38	1.2087	1	0.71176	0.34002	1	1	0.03401
Valine, leucine and isoleucine degradation	40	1.2724	1	0.73027	0.31434	1	1	0
N-Glycan biosynthesis	41	1.3042	1	0.73908	0.30235	1	1	0
Tryptophan metabolism	41	1.3042	1	0.73908	0.30235	1	1	0.14305

Supplementary table 4: Pathway analysis in CA3 using MetaboAnalyst

**Column1**	**Total**	**Expected**	**Hits**	**Raw p**	**-logP**	**Holm adjust**	**FDR**	**Impact**
Arginine biosynthesis	14	0.44533	4	0.000715	7.2436	0.060039	0.034261	0.22843
Purine metabolism	66	2.0994	8	0.000816	7.1114	0.067706	0.034261	0.13571
Alanine, aspartate and glutamate metabolism	28	0.89066	5	0.001494	6.5066	0.12248	0.041821	0.3125
Pantothenate and CoA biosynthesis	19	0.60437	4	0.00246	6.0074	0.1993	0.051669	0.17857
Aminoacyl-tRNA biosynthesis	48	1.5268	5	0.016121	4.1276	1	0.27083	0.16667
Taurine and hypotaurine metabolism	8	0.25447	2	0.024554	3.7069	1	0.33038	0.42857
Amino sugar and nucleotide sugar metabolism	37	1.1769	4	0.027531	3.5924	1	0.33038	0.07631
Phenylalanine metabolism	12	0.38171	2	0.05338	2.9303	1	0.52898	0.40476
Glutathione metabolism	28	0.89066	3	0.056676	2.8704	1	0.52898	0.26648
Glycine, serine and threonine metabolism	34	1.0815	3	0.090758	2.3996	1	0.76237	0.23069
Phenylalanine, tyrosine and tryptophan biosynthesis	4	0.12724	1	0.12141	2.1086	1	0.85184	0
Citrate cycle (TCA cycle)	20	0.63618	2	0.13101	2.0325	1	0.85184	0.06254
beta-Alanine metabolism	21	0.66799	2	0.14197	1.9521	1	0.85184	0
Sphingolipid metabolism	21	0.66799	2	0.14197	1.9521	1	0.85184	0.0142
Thiamine metabolism	7	0.22266	1	0.20287	1.5952	1	1	0
Valine, leucine and isoleucine biosynthesis	8	0.25447	1	0.22835	1.4769	1	1	0

Ascorbate and aldarate metabolism	10	0.31809	1	0.27693	1.284	1	1	0
Cysteine and methionine metabolism	33	1.0497	2	0.28285	1.2629	1	1	0.11776
Glycerophospholipid metabolism	36	1.1451	2	0.31879	1.1432	1	1	0.12872
Pyrimidine metabolism	39	1.2406	2	0.35435	1.0375	1	1	0.0811
Glycosylphosphatidylinositol (GPI)-anchor biosynthesis	14	0.44533	1	0.36527	1.0071	1	1	0.00399
Butanoate metabolism	15	0.47714	1	0.38565	0.95282	1	1	0
Nicotinate and nicotinamide metabolism	15	0.47714	1	0.38565	0.95282	1	1	0
Tyrosine metabolism	42	1.336	2	0.3893	0.94341	1	1	0.0254
Histidine metabolism	16	0.50895	1	0.40539	0.90291	1	1	0
Selenocompound metabolism	20	0.63618	1	0.47832	0.73748	1	1	0
Pentose phosphate pathway	21	0.66799	1	0.49513	0.70293	1	1	0
Pyruvate metabolism	22	0.6998	1	0.51142	0.67056	1	1	0
Propanoate metabolism	23	0.73161	1	0.52719	0.64019	1	1	0
Galactose metabolism	27	0.85885	1	0.58544	0.53539	1	1	0.0338
Glyoxylate and dicarboxylate metabolism	32	1.0179	1	0.64844	0.43318	1	1	0.04233
Arginine and proline metabolism	38	1.2087	1	0.71176	0.34002	1	1	0
Valine, leucine and isoleucine degradation	40	1.2724	1	0.73027	0.31434	1	1	0.02264
Primary bile acid biosynthesis	46	1.4632	1	0.77909	0.24963	1	1	0.02239

Supplementary table 5: Pathway analysis comparison between CA1 and CA3 for the top enriched pathways

Purine metabolism								
**Compound**	**Fold change CA1/CA3 at 1h**	***p* value at 1h**	**Fold change CA1/CA3 at 6h**	***p* value at 6h**	**Fold change CA1/CA3 at 24h**	***p* value at 24h**	**Fold change CA1/CA3 at 48h**	***p* value at 48h**
Xanthine	1.7588	0.0010891*	1.4582	0.29148	1.2818	0.21123	0.96751	0.95765
Adenosine	1.9538	0.0011372*	1.6099	0.18632	1.0913	0.69475	1.0978	0.87087
Hypoxanthine	1.2007	0.12287	0.94336	0.82242	1.0833	0.49377	1.0593	0.92024
Inosine	1.3733	0.16827	0.95208	0.86442	1.1782	0.42174	1.0969	0.88289
Guanosine	3.1701	0.014953*	1.5817	0.29517	1.387	0.4335	1.0583	0.95733
P1,P4-Bis(5'-xanthosyl) tetraphosphate	0.51471	0.012739*	0.5637	0.03795 *	0.66243	0.12286	0.58191	0.20417

Adenine	1.7042	0.0026681*	1.4266	0.27022	1.0774	0.68995	1.0837	0.87641
Xanthylic acid	1.1021	0.16955	0.92621	0.78003	0.91277	0.38845	0.90798	0.87198
5-Aminoimidazole ribonucleotide	0.73986	0.1403	0.65573	0.14012	1.0474	0.79435	0.75144	0.6688
Deoxyadenosine	2.29	0.0438*	2.4508	0.12075	0.69645	0.47698	1.6237	0.27851
D-ribulose-5-phosphate	1.3824	0.16744	1.0421	0.88944	0.99669	0.98669	1.0749	0.90482

Pantothenate and CoA biosynthesis

**Compound**	**Fold change CA1/CA3 at 1h**	***p* value at 1h**	**Fold change CA1/CA3 at 6h**	***p* value at 6h**	**Fold change CA1/CA3 at 24h**	***p* value at 24h**	**Fold change CA1/CA3 at 48h**	***p* value at 48h**
4-Phosphopantothenoylcysteine	1.6829	0.064049	1.643	0.26622	0.99152	0.98036	1.2038	0.7434

Cysteine	1.4951	0.11354	1.1476	0.63446	1.0804	0.60779	0.98479	0.97766
Aspartic acid	1.2117	0.17311	0.95263	0.85261	1.0316	0.81506	0.96321	0.94597
Uracil	1.4419	0.13372	0.92985	0.77577	0.88862	0.71559	0.83425	0.70403
**Alanine, aspartate and glutamate metabolism**								
**Compound**	**Fold change CA1/CA3 at 1h**	***p* value at 1h**	**Fold change CA1/CA3 at 6h**	***p* value at 6h**	**Fold change CA1/CA3 at 24h**	***p* value at 24h**	**Fold change CA1/CA3 at 48h**	***p* value at 48h**
Succinic acid	0.62752	0.01464*	0.64719	0.051904	1.0038	0.96565	0.76293	0.56864
Fumaric acid	1.2355	0.0083438 *	0.91613	0.72676	1.0114	0.80613	1	1.0
Aspartic acid	1.2117	0.17311	0.95263	0.85261	1.0316	0.81506	0.96321	0.94597
Alanine	1.0263	0.3037	0.84792	0.49557	0.96783	0.44661	0.92894	0.89177
N-acetyl-L-aspartic acid	1.0206	0.73904	0.94738	0.83424	1.0058	0.96128	1.1	0.87343
**Amino sugar and nucleotide sugar metabolism**								
**Compound**	**Fold change CA1/CA3 at 1h**	***p* value at 1h**	**Fold change CA1/CA3 at 6h**	***p* value at 6h**	**Fold change CA1/CA3 at 24h**	***p* value at 24h**	**Fold change CA1/CA3 at 48h**	***p* value at 48h**

UDP-N-acetylhexosamine	2.014	0.039048 *	1.3797	0.56825	0.69802	0.58776	0.88629	0.85069
Cytidine-5'-monophosphate-5-N-acetylneuraminic acid	1.2751	0.19647	1.0594	0.47213	1.2953	0.29737	1.3966	0.49665
N-acetylhesoxamine 6-phosphate	1.1604	0.51195	0.6991	0.1415	0.92597	0.59162	0.84709	0.78809
Hexose 1-phosphate	1.2324	0.66317	0.57718	0.10502	0.93001	0.88252	0.96336	0.95602

Arginine Biosynthesis

**Compound**	**Fold change CA1/CA3 at 1h**	***p* value at 1h**	**Fold change CA1/CA3 at 6h**	***p* value at 6h**	**Fold change CA1/CA3 at 24h**	***p* value at 24h**	**Fold change CA1/CA3 at 48h**	***p* value at 48h**
Fumaric acid	1.2355	0.0083438 *	0.91613	0.72676	1.0114	0.80613	1	1
Aspartic acid	1.2117	0.17311	0.95263	0.85261	1.0316	0.81506	0.96321	0.94597
N-acetylornithine	1.1319	0.10318	0.87532	0.58444	0.99968	0.9983	1.1535	0.80676
Citrulline	0.95223	0.57081	0.66935	0.093512	0.89638	0.27443	1.0666	0.90996
Arginine	1.1262	0.36643	0.73376	0.21789	1.0314	0.75544	0.83497	0.72117

Aminoacyl tRNA biosynthesis

**Compound**	**Fold change CA1/CA3 at 1h**	***p* value at 1h**	**Fold change CA1/CA3 at 6h**	***p* value at 6h**	**Fold change CA1/CA3 at 24h**	***p* value at 24h**	**Fold change CA1/CA3 at 48h**	***p* value at 48h**
Cysteine	1.4951	0.11354	1.126	0.70198	1.126	0.70198	0.98297	0.98046

L-phenylalanine	1.2398	0.30231	0.91307	0.77351	0.91307	0.77351	1.2577	0.71714
Tryptophan	1.1845	0.45237	0.92792	0.78877	0.92792	0.78877	1.2041	0.76089
Aspartic acid	1.1466	0.0020419	0.93421	0.77241	0.93421	0.77241	0.9491	0.91734
Arginine	1.1262	0.36643	0.73411	0.21776	0.73411	0.21776	0.83498	0.72114
Serine	1.0999	0.17174	0.84517	0.49276	0.84517	0.49276	0.93258	0.89637
Leucine	1.0979	0.58169	0.98114	0.93911	0.98114	0.93911	1.1442	0.79511
L-Threonine	1.0546	0.5682	0.83023	0.45916	0.83023	0.45916	1.0187	0.97277
L-histidine	1.0426	0.84052	0.74632	0.27004	0.74632	0.27004	0.95434	0.92202
**Pyrimidine metabolism**								
**Compound**	**Fold change CA1/CA3 at 1h**	***p* value at 1h**	**Fold change CA1/CA3 at 6h**	***p* value at 6h**	**Fold change CA1/CA3 at 24h**	***p* value at 24h**	**Fold change CA1/CA3 at 48h**	***p* value at 48h**
Cytidine	1.4302	1.5202E-6*	1.014	0.95781	0.94387	0.59865	1.2009	0.76026
Cytidine-5'-diphosphate (CDP)	1.0789	0.47721	1.0338	0.50484	1.175	0.15044	1.1046	0.79249
dCMP	1.315	0.25596	1.3745	0.58606	1.1696	0.63434	0.80876	0.76938
Uracil	1.4419	0.13372	0.92985	0.77577	0.88862	0.71559	0.83425	0.70403

Uridine	1.3193	0.019158*	0.97654	0.93243	1.0135	0.91674	1.0344	0.95344
**Beta-Alanine metabolism**								
**Compound**	**Fold change CA1/CA3 at 1h**	***p* value at 1h**	**Fold change CA1/CA3 at 6h**	***p* value at 6h**	**Fold change CA1/CA3 at 24h**	***p* value at 24h**	**Fold change CA1/CA3 at 48h**	***p* value at 48h**
Uracil	1.4419	0.13372	0.92985	0.77577	0.88862	0.71559	0.83425	0.70403
Aspartic acid	1.1466	0.0020419	0.93421	0.77241	0.93421	0.77241	0.9491	0.91734
L-histidine	1.0426	0.84052	0.74632	0.27004	0.74632	0.27004	0.95434	0.92202
**Taurine and hypotaurine metabolism**								
**Compound**	**Fold change CA1/CA3 at 1h**	***p* value at 1h**	**Fold change CA1/CA3 at 6h**	***p* value at 6h**	**Fold change CA1/CA3 at 24h**	***p* value at 24h**	**Fold change CA1/CA3 at 48h**	***p* value at 48h**
Cysteine	1.4951	0.11354	1.126	0.70198	1.126	0.70198	0.98297	0.98046
Taurine	1.1	0.007*	0.909	0.704	1.005	0.899	1.136	0.797

Legend: Asterisk: statistically significant
